# Glycidyl Azide Polymer and its Derivatives-Versatile Binders for Explosives and Pyrotechnics: Tutorial Review of Recent Progress

**DOI:** 10.3390/molecules24244475

**Published:** 2019-12-06

**Authors:** Tomasz Jarosz, Agnieszka Stolarczyk, Agata Wawrzkiewicz-Jalowiecka, Klaudia Pawlus, Karolina Miszczyszyn

**Affiliations:** Department of Physical Chemistry and Technology of Polymers, Silesian University of Technology, 9 Strzody Street, 44-100 Gliwice, Poland

**Keywords:** glycidyl azide polymer, energetic binder, propellant, plastic-bonded explosive, curing, plasticizer

## Abstract

Glycidyl azide polymer (GAP), an energetic binder, is the focus of this review. We briefly introduce the key properties of this well-known polymer, the difference between energetic and non-energetic binders in propellant and explosive formulations, the fundamentals for producing GAP and its copolymers, as well as for curing GAP using different types of curing agents. We use recent works as examples to illustrate the general approaches to curing GAP and its derivatives, while indicating a number of recently investigated curing agents. Next, we demonstrate that the properties of GAP can be modified either through internal (structural) alterations or through the introduction of external (plasticizers) additives and provide a summary of recent progress in this area, tying it in with studies on the properties of such modifications of GAP. Further on, we discuss relevant works dedicated to the applications of GAP as a binder for propellants and plastic-bonded explosives. Lastly, we indicate other, emerging applications of GAP and provide a summary of its mechanical and energetic properties.

## 1. Introduction

**Figure 1:**
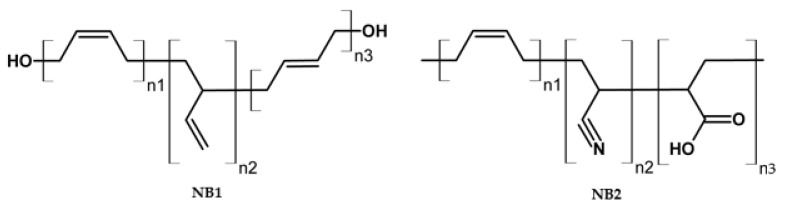
Chemical structures of non-energetic binders commonly used for the production of propellants.

Solid propellant [1] and explosive [2] formulations both include a relatively small amount of a natural or synthetic polymer. This polymeric additive acts as a binder, improving cohesion and granting the propellant or explosive favourable mechanical properties. Although preventing the formulation from breaking or cracking when subjected to mechanical stress is the primary function of a polymeric binder, it can have other secondary functions, e.g., promoting adhesion between a propellant and the inner lining of a rocket engine and assuring steady combustion of the fuel. Another aspect, which can be easily overlooked, is the sensitivity of the binder to different initiating stimuli. Similarly, the combustion parameters of the binder should also be evaluated because they can significantly affect the properties of the entire formulation, even despite the low content of the binder (well-below 30% in a vast majority of reported formulations). In this aspect, the differences between non-energetic and energetic polymer binders are the most pronounced. Non-energetic polymers, such as hydroxyl-terminated polybutadiene (HTPB, **NB1**) [3] and butadiene-acrylonitrile-acrylic acid terpolymer (PBAN, **NB2**) [4] (Figure 1), typically undergo endothermic decomposition, significantly lowering the net combustion heat of the formulation, while energetic polymers release heat during combustion, often comparably so to the other components of the formulation [5].

Although the above questions and issues are the ones that may first come to mind, when considering the binder for a particular solid propellant or polymer-bonded explosive (PBX) system, the design and selection of a binder is a non-trivial process that involves numerous other factors [6]. This is because both the performance of the binder in a formulation and any additional properties it may bestow depend on the nature of interactions and despite the relatively low reactivity of most polymeric species, even chemical reactions with the other components of that formulation. Consequently, extensive experimental investigation is essential to make the best choice of binder for a particular formulation and often can reveal unexpected properties of that formulation. This experimental support typically involves thermal Differential Scanning Calorimetry (DSC), Differential Thermal Analysis (DTA), Thermogravimetry (TG), mechanical (determination of stress-strain curves and Young’s modulus), sensitivity (primarily friction and impact sensitivity, but also electrostatic discharge and shock sensitivity) and combustion (typically profiles of combustion rate vs. binder content or vs. oxidising agent/fuel content) studies and a significant share of works published about each binder are dedicated to providing an extremely detailed picture of its properties and interactions with various other materials.

Another area of research interest is of course the application of the binders in particular formulations and their performance. Although properties such as sensitivity are commonly reported, the main emphasis is on the properties specific for a given class of applications, such as changes in the detonation parameters of the main explosive (negligible changes are highly desirable) caused by the binder, in the case of PBXs, or specific impulse, in the case of propellants. Stemming from the two main subjects of published reports is the area of modifying the properties of binders and tuning them for particular applications. Depending on how commonly used, well-investigated and versatile a particular binder is, one of the above research areas will be more prominent and others may be only scarcely represented.

Glycidyl azide polymer (GAP) is a well-known and well-investigated system (typical properties of commercially available GAP-polyol are shown in Table 1) that has been in widespread application for almost three decades, leaving little possibility for discovering any novel properties or features. Consequently, the vast majority of recent research works, dedicated to GAP, can be classified as attempts at optimising the synthetic procedure, fine-tuning the properties of the binder, via structural modifications, or developing new processing methods for application in particular formulations. In light of this, we opted to sort such recent developments into two main categories – synthetic, focusing on the structural and additive-based modifications of GAP, and applicatory, focusing on adapting GAP to particular formulations and optimising the performance of GAP-containing formulations.

## 2. Synthesis, Modification and Properties of GAP

The typical synthetic procedure for obtaining GAP or other azide-bearing polymers consists of the polymerisation of an azide-free monomer and its subsequent functionalization with azide groups (azidation). This is because of the difficulty in achieving polymerisation of azide-bearing monomers, such as glycidyl azide [8]. Although a number of substrates, such as poly (propylene glycol), polyglycidol or polyepichlorohydrin (PECh) can be azidated to produce GAP, industrial production relies on polyepichlorohydrin, as the most favourable substrate.

**Scheme 1:**
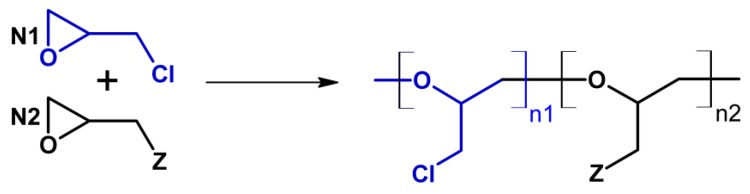
Example of pre-azidation structural modification: copolymerisation of epichlorohydrin (blue) and another epoxide-based co-monomer, bearing a functional group Z.

Structural modification can be readily carried out even prior to the azidation stage, by substituting PECh with a copolymer of epichlorohydrin, whose co-monomers may or may not undergo azidation (Scheme 1). 

**Scheme 2:**
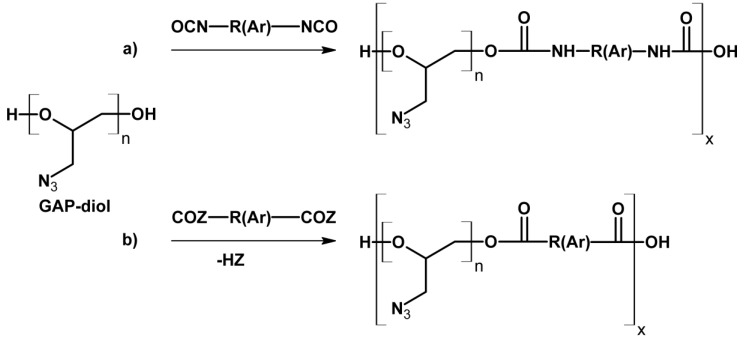
Examples of polyreactions utilising the terminal hydroxyl groups of GAP-diol; (**a**) synthesis of polyurethanes; (**b**) synthesis of polyesters (Z = Cl, OH); R(Ar) denotes an aliphatic or aromatic moiety; chemical formulae of commonly used curing agent structures are shown in Figure 4.

Assuming that standard GAP is synthesised, a hydroxyl-terminated oligomer or polymer is obtained. Depending on whether the product is linear or branched, it is dubbed GAP-diol or GAP-polyol (most commonly GAP-tetraol) respectively. Due to the presence of the reactive hydroxyl groups, these products can be used for further polyreactions, potentially yielding polyurethanes, polyesters or other types of derivatives (Scheme 2), offering a large degree of freedom in designing the structure of a GAP-bearing end-polymer. By using another hydroxyl-terminated (macro)molecule alongside GAP, even wider modification possibilities are made available.

**Scheme 3:**
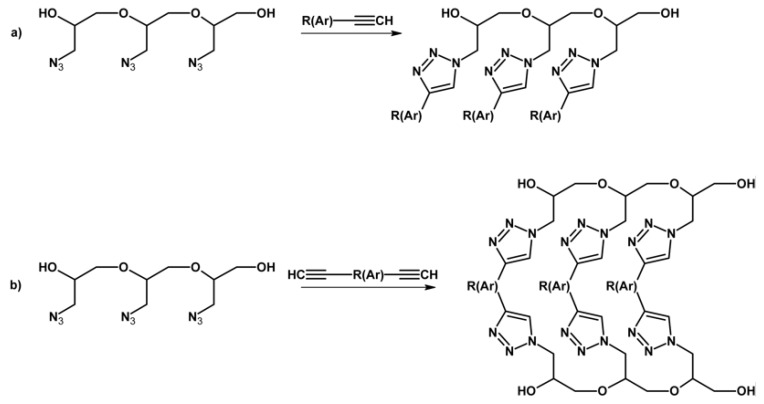
Synthesis of triazole moieties through the azide-alkyne click reaction, using GAP (GAP trimer is depicted for clarity): (**a**) azide group transformation—a monofunctional alkyne is used; (**b**) curing—a difunctional alkyne reagent is used. R(Ar) denotes an aliphatic or aromatic moiety; chemical formulae of commonly used curing agent structures are shown in Figure 3.

Rather than only using the hydroxyl groups, the azide groups of GAP can also be used for post-synthesis modification of the polymer, as described in Section 2.2 and Section 2.4. The most common of those methods is the use of a click chemistry reaction between the azide groups of the modified polymer and alkyne-bearing species, producing a triazole ring linking group (Scheme 3). It is worth noting that due to steric hindrance, not all azide groups will necessarily undergo the reaction.

A good review of the various methods of synthesising GAP and GAP-based copolymers was recently published by Eroglu and Bostan [9], with the authors detailing a number of synthetic procedures; as such, herein we are reporting only the most recent advances in this subject.

Although only focusing on the introduction of poly (ethylene glycol) substituents into GAP, a review by Ikeda [10] gives a good overview of the practical aspects of the azide-alkyne reaction, as well as providing an insight into the potential applications of such GAP derivatives.

### 2.1. Recent Trends in the Synthesis of Glycidyl Azide Polymers

Attempts at optimising the procedure of synthesising GAP are relatively rare among recent works, as the process has been extensively explored in the past. Even so, improvements continue to be implemented as, due to economies of scale, even gains considered too minor to be of interest, in the case of a laboratory-scale synthesis, can translate into significant economic benefits on an industrial scale. Similarly, if one reagent were to be substituted by another, with a lower molecular weight, it would be of little to no interest at the laboratory scale, provided the yield of the end product did not change. At the industrial scale, however, such an apparently trivial optimisation can mean a difference of hundreds of kilograms being pumped from reactor to reactor, through a facility and eventually needing to be recycled. A similar paradigm shift is needed when considering the choice of reaction medium—both in terms of operations (transport, but also heating or cooling) on large volumes and in terms of dealing with potentially harmful or toxic sewage. As such, the works highlighted in this section are best evaluated from a more practical viewpoint, taking the abovementioned issues into consideration.

**Figure 2:**
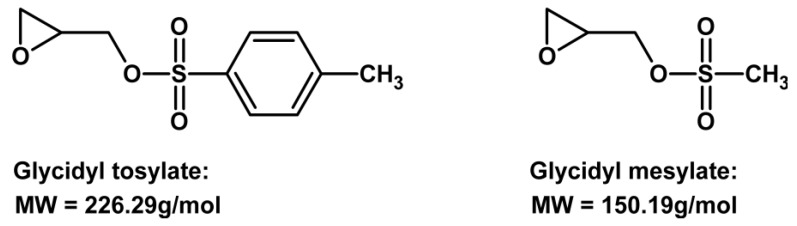
Structures of glycidyl tosylate and glycidyl mesylate, used to prepare GAP via polymerisation and azidation [10]. Molecular weights of the two species are shown for comparison.

An interesting modification of the “standard” synthetic procedure is to use of polymers bearing mesylate groups rather than tosylate groups for the azidation stage [11]. Mura et al. tested this approach to the syntheses of GAP and poly 3-azidomethyl-3-methyl oxetane (PAMMO), using procedures involving tosylate intermediates [12,13] as standards, against which their modified procedures were compared. Azidation of both the mesyl and tosyl intermediates is reported to proceed quantitatively, for procedures yielding both GAP and PAMMO. Studies of the kinetics of azidation of mesylate- and tosylate-bearing precursors show comparable performance, although a beneficial effect of the higher mobility of the mesylate group on the azidation reaction is seen. An interesting point, made by the Authors, is that, although the two intermediates are comparable on a laboratory scale, the use of tosylate on an industrial scale (due to the large molecular weight of the group in comparison with the mesylate group, Figure 2) necessitates the use and handling of a significantly larger mass of reagents and by-products to produce a unit mass of either GAP or PAMMO than would be required for a mesylate-mediated process.

Rather than focusing on the choice of intermediates bearing chloride, tosylate or mesylate groups for the azidation stage of GAP synthesis, Xu et al. [14] investigated the potential of a water/ionic liquid binary system as the reaction medium. The lack of organic solvents used in this procedure is an important advantage, making it “green”, as the authors point out. Although the yield of the reaction varied greatly with the composition of the binary solvent system, the authors have managed to find an apparently optimal composition and achieved a yield of 89%, significantly higher than what was achieved for either of the pure solvents (0% and 50% for water and the ionic liquid respectively). Interestingly, the molecular weight of the produced GAP was in the range of 3300 ÷ 3600 g/mol, regardless of the composition of the reaction medium.

Grinevich et al. [15] have also approached this synthesis, instead utilising phase transfer catalysis for the azidation of several types of epichlorohydrin oligomers and polymers. Although the synthetic procedure still requires elevated temperature, the Authors were able to significantly lower the time necessary for obtaining the products and managed to circumvent the potential problem of removing non-volatile solvents from the obtained raw GAP. Relatively high yields (87 ÷ 97%) were obtained when oligomeric ethers were used for the reaction and moderate yields (up to 60%) when macrocyclic ethers were used instead.

Lastly, an interesting variation on the synthesis of GAP leads to a system terminated with four hydroxyl groups (GAP-tetraol). This approach was reported by Soman et al. [16], who developed it using a polyepichlorohydrin-diol in order to improve the curing kinetics of GAP for use as an energetic binder. Investigation of the product revealed that the thermal properties of the GAP-tetraol are similar to those of the typical GAP; the GAP-tetraol was also found to be more viscous and have a slightly extended viscoelastic range than the typical GAP. Interestingly, when the two GAP systems are subjected to isocyanate curing agents, significant discrepancies are observed – the curing reaction is much faster for GAP-tetraol, allowing it to be cured with less reactive curing agents than those required for curing typical GAP.

### 2.2. Modification of GAP through Curing

**Figure 3:**
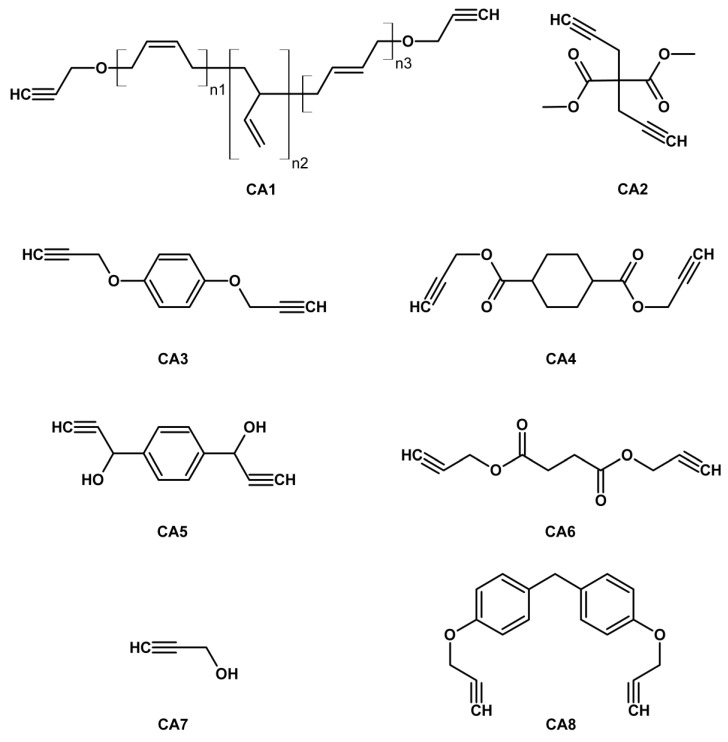

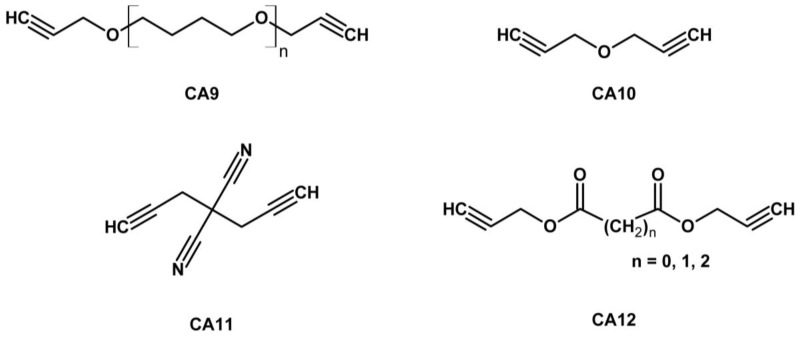
Chemical structures of alkyne-bearing curing agents.

**Figure 4:**
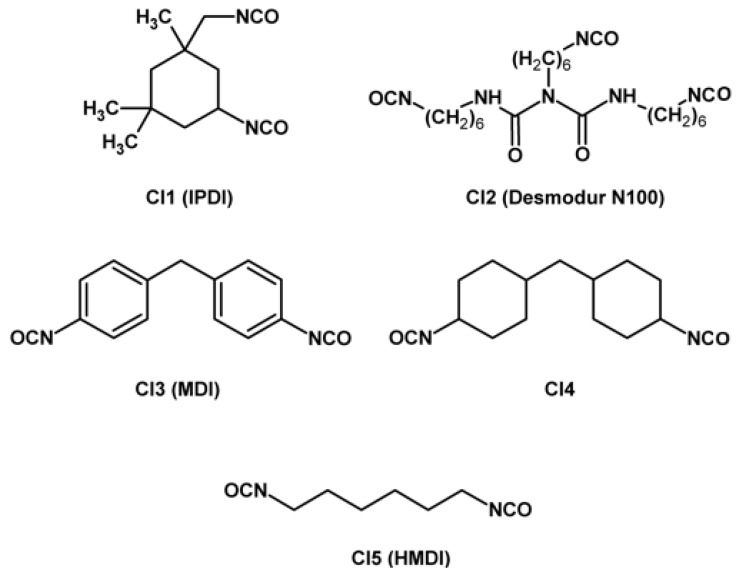
Chemical structures of isocyanate-bearing curing agents. Common names are given in parentheses.

The commercially available GAP is typically a liquid and, as such, has no mechanical strength to speak of. Although special, high molecular weight fractions of GAP may be solid, curing is required for the polymer to actually enhance the mechanical properties of any formulation it is used in. Two types of functional groups are present in the macromolecule (hydroxyl and azide) and can be exploited for curing the polymer; in both cases the process is readily accomplished due to the high reactivity of these groups (Figure 3 and Figure 4). Unlike the liquid GAP, the cured macromolecule is a solid and typically shows favourable mechanical properties, similar to analogous cured poly (alkylene oxides). The changes, effected in the mechanical and thermal properties of GAP during curing, are dependent primarily on the choice of curing agent (chemical structure and functionality) and the curing density (arising from the GAP/curing agent ratio in the feed for the curing reaction); these are, however, not the only relevant factors, as reported below.

Ding et al. [17] report an interesting variation of the azide-alkyne reaction. Rather than curing GAP with a short alkyl or aromatic agent, the authors employed a polymeric curing agent, propargyl-terminated polybutadiene (**CA1**). The authors varied the amount of the curing agent used, finding the most favourable mechanical properties for systems with a 2:1 azide to alkyne functional group ratio.

Building on the above report, Hu et al. [18] investigated the properties of GAP alkyne-cured by dimethyl 2,2-di(prop-2-ynyl)malonate (**CA2**), in order to improve the loading level of GAP, due to the substitution of the polymeric curing agent with a small molecular agent. Similarly to the abovementioned report, the authors investigated the properties of systems produced from different GAP/curing agent ratios, finding, unlike the previous work, that increasing the cross-linking density yielded consistent increases of the Young’s modulus and tensile strength of the materials, while, expectedly, lowering their ability to undergo swelling.

A similar approach is reported by Sonawane et al. [19], who cured GAP using bispropargylhydroquinone (**CA3**). Interestingly, the authors studied not only the mechanical properties of the cured polymer, but also investigated the activation energy of its decomposition, although it is unclear which GAP/curing agent ratio was used for this experiment. The Authors [20] have also investigated a wide array of bispropargyl derivatives as potential curing agents for GAP, focusing on the kinetics of the process and attempted to explain the reactivity of such species towards the azide groups of GAP, using quantum-chemical simulation [21] to provide a theoretical framework to their earlier works.

A novel approach to the use of azide-bearing polymers as solid propellants was reported by Li et al. [22], who treated GAP and poly(3-azidomethyl-3-methyl oxetane) (PAMMO) with a difunctional curing agent (**CA4)**. The curing process was realised through a reaction between the alkyne groups of the curing agent and the azide groups of the polymers, producing triazole bridges between individual polymer chains. Cross-linking was found to improve the mechanical performance of the two polymers, with their properties being a function of the cross-linking density. Although both cured polymer systems were found to exhibit improved properties, the improvement was more pronounced for systems based on PAMMO, showing higher tensile strengths and elongations at break than GAP-based systems with a comparable cross-linking density. The curing process itself was also studied and in both cases, was found to conform to a first-order kinetic model.

Another approach to modifying the properties of GAP, reported by Tanver et al. [23] relies on preparing an interpenetrating polymer network (IPN), using acyl-terminated GAP and HTPB. These two systems are simultaneously cured, the former through the use of an alkyne-bearing curing agent (**CA2**) and the latter by a reaction with an isocyanate curing agent (**CI1**). In their work, the authors synthesised IPNs with different initial acyl-terminated GAP contents, with the most favourable set of thermal and mechanical properties (tensile strength of 5.26 MPa and a 318% elongation at break) being found for a 1:1 (*w*/*w*) mixture of HTPB/acyl-GAP.

In their later work, the authors [24] substituted the acyl-terminated GAP and HTPB frameworks with GAP-multi-walled carbon nanotube (MWCNT) and HTPB-MWCNT frameworks, respectively, taking advantage of the mechanical properties of nanotubes. The nanotube-functionalised polymers were mixed together and cured (**CI1**, **CI2**) to produce the interpenetrating polymer network architecture, with the authors investigating different GAP-MWCNT/HTPB-MWCNT ratios. The constituents themselves and the final IPN were found to be insensitive to impact (>40 J), friction (>360 N) and electrostatic discharge (>5 J). Composite propellants using these binders were also tested; their exact composition is however unclear. Expectedly, the shift from cured GAP to the IPN architecture resulted in a large improvement in terms of mechanical properties, with an 8.17 MPa tensile strength and 312% elongation at break being found for an IPN containing a 50:50 ratio of GAP/MWCNT and HTPB/MWCNT.

The same authors later explored the subject of utilising polymer networks as means for improving the mechanical properties of propellants [25], reporting an energetic polyurethane, prepared through sequential curing of GAP and HPTB (**CI1**, **CI2**). Similarly to their earlier study, the authors investigated networks produced using different ratios of the two prepolymers, finding that a 1:1 GAP/HTPB ratio yields the most favourable mechanical properties, providing another promising system for use in propellants.

A different method of producing GAP/carbon nanotube composites is reported by Wang et al. [26] who cross-linked GAP with a propargyl-terminated polymeric curing agent (**CA9**) in MWCNT and carboxyl-functionalised MWCNT dispersions. As such, the authors chose to rely on occlusion as means of “trapping” the nanotubes in the solidifying cross-linked polymer matrix rather than using direct chemical linkage, as in the abovementioned case of GAP-MWCNT/HTPB-MWCNT networks. The different MWCNT trapping mechanism is well reflected in the mechanical properties of the composite, exhibiting, for carboxyl-functionalised MWCNTs, a tensile strength of 1.41 MPa and Young’s modulus value of 6.33 MPa, implying that while the nanotubes promote stiffness, they do not significantly increase the resilience of the composite, possibly due to relatively scarce MWCNT/GAP interactions. 

**Figure 5:**
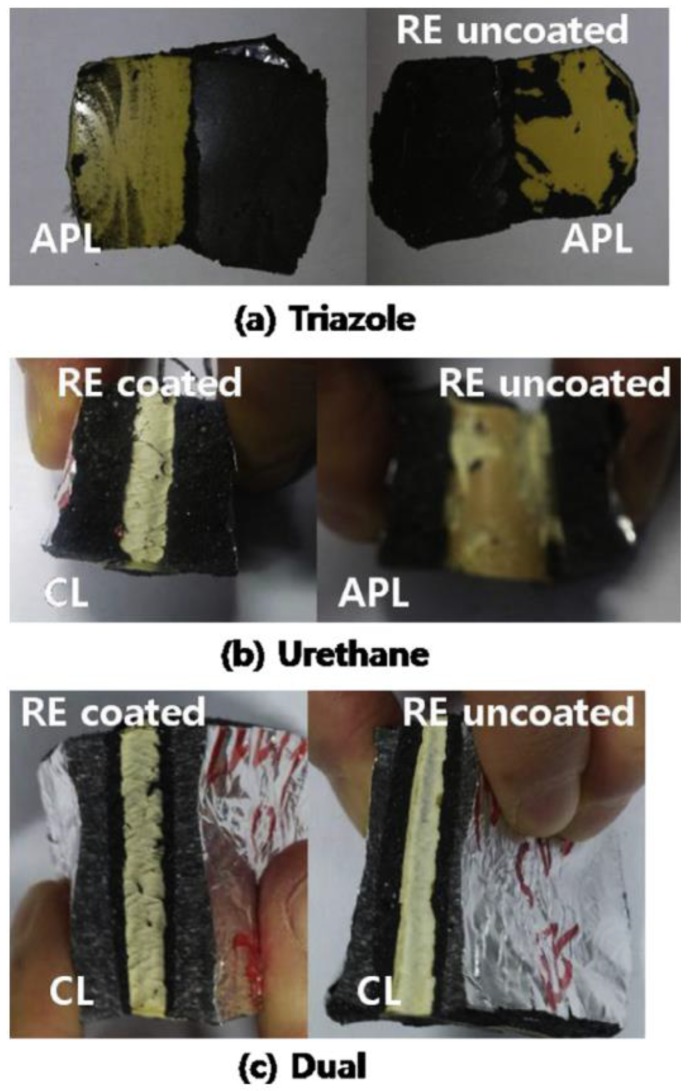
Photographs of liner/propellant cross-sections for GAP/AP/HMX solid composite propellants prepared using different curing systems (black: propellant, yellow: liner): (**a**) curing via azide groups, forming a triazole moiety; (**b**) curing via hydroxyl groups, forming a urethane moiety; (**c**) curing via both azide and hydroxyl groups. Indications: “APL (adhesive propellant/liner) indicates that the break showed no evident mark of either the propellant or the liner on the opposite surface; CL (cohesive in liner) indicates that break took place within the liner” [27].

Next, Min et al. [27] report an interesting approach to curing GAP, simultaneously utilising both potential curing pathways, by utilising difunctional isocyanate (**CI1**, **CI2**) and difunctional alkyne curing agents (**CA5, CA6**), producing urethane and triazole linkers. The “dual-cured” GAP was both tested by itself and used to prepare propellants, whose mechanical properties were subsequently determined, revealing, for a GAP/AP/HMX propellant, significant improvement in comparison with propellants based on GAP cured by either of the two pathways. In the case of a more elaborate GAP/RDX/HMX/CL-20 propellant, the dual-cured GAP showed performance inferior to purely polyurethane-cured GAP, however, substituting the isocyanate curing agent with isocyanate-terminated GAP yielded a propellant formulation with superior mechanical properties (Table 5). An interesting highlight of the report is the study of the adhesion of the propellants to an elastomer membrane, imitating the liners used in rockets and missiles, crucial for any widespread applications. In this aspect, the dual-cured GAP excels, with the propellants based on it showing far better adhesion to the liner than those based on either of the single-cured GAP systems (Figure 5).

Hagen et al. [28] report an interesting experimental comparison of different methods for curing GAP, i.e., isocyanate curing (**CI1**, **CI2**), isocyanate-free (alkyne) curing (**CA3**, **CA5–8**), simultaneous dual curing and sequential dual curing. The produced systems are evaluated in terms of their mechanical properties. It is worth noting that the authors included mixed isocyanate curing agents in the comparison; the use of such agents is known to yield more favourable properties of the cured systems than in the case of curing using a single isocyanate agent. An interesting feature of the article is the choice of the non-isocyanate curing agent for the sequential curing, as the alkyne-bearing agent was equipped with a hydroxyl group, whose inclusion into the cured GAP produced additional reactive sites for the subsequent curing with the isocyanate-bearing agent. Mechanical studies revealed that although simultaneous dual curing yielded generally more favourable properties than alkyne curing, better performance was achieved using mixed isocyanate curing. Even so, the best properties were found for sequential curing, supplemented by improved control over the process and process safety, due to the separation of the two curing stages.

In the recent work of Araya-Marchena [29], the authors investigated the use of dialkynyls for curing azide-bearing polymers via a click chemistry reaction. To maximise the energetic content of the cured polymer, dialkynyls of relatively small molecular weight were chosen, i.e., bis-propargyl ether (**CA10**), 4,4’-dicyanohepta-1,6-diyne (**CA11**) and three bis-propargyl esters (**CA12**): bis-propargyl oxalate, malonate, succinate. The authors described curing reaction kinetics and energetics (activation energy, reaction order and reaction enthalpy) depending on the dialkynyl concentration in the mixture, which may allow using these dialkyne compounds on a larger scale by making it possible to predict the behaviour of the reacting mixtures and safely control their exothermic curing process.

**Figure 6:**
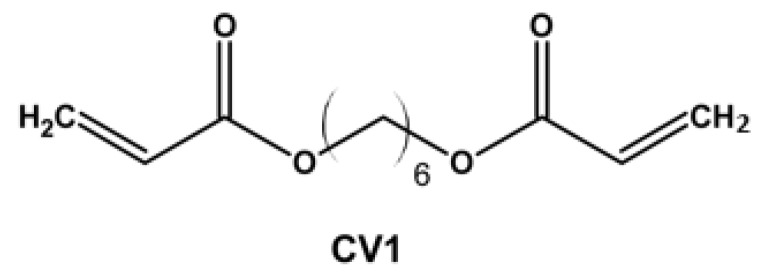
Chemical structure of a vinyl-bearing curing agent.

Another approach to curing GAP was adopted by Agawane et al. [30], who employed multiple curing agents: mixtures of di- (**CI1**, **CI3**) and tri-functional (**CI2**) isocyanates, an alkyne curing agent (**CA6**) and, most interestingly, an acrylate-based (**CV1**) curing agent (Figure 6). Although the authors, similarly to Hagen [28], achieved the best mechanical properties when using a mixture of isocyanate curing agents, they did not attempt sequential curing, which may have resulted in even better properties. It is worth noting that the mixed isocyanate-cured GAP burns smoothly, with 50 g of the cured polymer being consumed in 7.5 s. However, few other experimental details are given for this investigation. Interestingly, heat outputs in the range of 4744 ÷ 5156 cal/g (19.8 ÷ 21.6 kJ/g) are reported for the cured polymers, much higher than for the non-energetic HTPB binder (below 0.05 kJ/g) [31,32].

Spherical GAP-based propellants are rather popular components of composite modified double base propellant formulations [33]. One of the ways to improve the performance of these formulations is to optimise the curing of the resin, a non-trivial task, requiring deep understanding of the process. In order to gain that comprehension, Wei et al. [34] investigated the curing kinetics of the spherical GAP propellant using rheometric methods. The curing experiment was carried out for a model system of GAP polyol, supplemented by Bu-NENA and isophoron diisocyanate (IPDI) curing agent (**CI1**), with the authors preparing the spherical GAP propellant themselves, using a GAP nitrocellulose ratio of 3:7. A different ratio was also investigated in the authors’ parallel work [35]. Interestingly, rather than investigating different compositions of the mixture, which would interfere with other components of the composite modified double base propellant, the authors focused on investigating the curing kinetics for different heating rates. By linking the degree of conversion in the curing reaction with the storage modulus, the authors were able to determine the kinetic parameters of the process, using isoconversional methods.

An interesting report by Deng et al. [36] details the effects of different bonding agents on the properties of GAP-based composite propellants. The authors applied these bonding agents (N,N’-bis(2-hydroxyethyl)-dimethylhydantoin, 1,3,5-trisubstituted isocyanurates, cyano- hydroxylated amines and a hyper-branched polyether with terminal groups substituted by hydroxyl, cyano and ester functional groups), as coatings on HMX and ammonium perchlorate grains, in order to improve their adhesion to cured GAP (**CI1**, **CI2**), which was used as an energetic binder. This approach proved successful, as even 0.3% (*w*/*w*) of the bonding agent was sufficient to increase the modulus of the propellant from 1.48 MPa to 2.26 MPa, while the elongation at break was roughly constant.

Adopting a more fundamental approach, Ma et al. [37] focused on the cross-linking GAP using toluene diisocyanate, investigating the effects of cross-linking density on the mechanical properties of the cured polymers. Although the cured polymer with the best properties showed a tensile strength of 1.6 MPa and a 1041% elongation at break, the Authors were able to gain valuable insights into the evolution of mechanical properties with increasing cross-linking density.

### 2.3. Development of GAP Derivatives and Plasticizers

Although energetic binders, exhibiting favourable mechanical properties, can be prepared by curing GAP, alternative approaches are also common. The most prominent alternative is to produce derivatives—GAP-based binders—through copolymerisation. This modification can be readily implemented at different stages of the synthetic procedure, by using a mixture of co-monomers to prepare the azide-free prepolymer (Figure 2) or by using a mixture of GAP and other (macro)co-monomers for follow-up polyreactions. In the latter case, the copolymerisation product is typically subjected to curing and often becomes extremely similar structurally to the macromolecules produced by curing GAP with the use of the more sophisticated curing agents, causing the two methods to converge in terms of the obtained product. An interesting feature of the methods involving copolymerisation is that they allow the topology of the product to be tailored, by involving synthetic procedures yielding ordered (i.e., block or multi-block, alternating or even graft) copolymers. The occurrence of such ordering in the binders may grant them a wide array of additional, beneficial properties, such as self-assembly capabilities (possibility of encapsulating components of a formulation) or microphase separation (formation of nanostructures, showing differing properties, potentially more compatible with different types of formulation components), in the case of block copolymers.

One particular drawback of GAP, despite the polymer being widely used as an energetic binder, is the fact that it loses elastomeric properties at temperatures below 6 °C. To remedy this and allow low temperature applications, copolymers of GAP and tetrahydrofuran (THF) or poly(ethylene glycol) (PEG) were developed [38,39]. Recently, a more environmentally-friendly method of preparing such copolymers was reported by Kshirsagar et al. [40], in which the azidation stage involves microwave irradiation. The authors optimised the conditions of the reaction and, with the correct choice of the reaction medium, achieved approx. 90% conversion at a temperature of 90 °C and reaction time of minutes, as opposed to the 120 °C and reaction time on the order of hours for the conventional synthesis method. An average molecular weight of 1320 g/mol is reported, found by Gel Permeation Chromatography (GPC). This is well within the 500–5000 g/mol range, required for most reported applications, although no mention has been made of the molecular weight standards used, the choice of which may affect the obtained value to a noticeable extent.

An approach similar to the above was also taken by Dong et al. [41], who prepared an azidated glycidyl-tetrahydrofuran copolymer, poly (glycidyl azide-r-3-azidotetrahydrofuran), envisioned as a potential energetic binder for solid propellants and intended to supplant GAP in this role. The structural identity of the copolymer was investigated and confirmed with a wide array of techniques; the friction, impact and electrostatic discharge sensitivities were also investigated, with the copolymer being insensitive to friction and impact, while showing a 181 mJ (E_50%_) sensitivity to electrostatic discharge.

Qui et al. [42] fabricated GAP-based composites with propargyl-terminated ethylene oxide-co-tetrahydrofuran copolymer (PPET) exhibiting two (p-) and three (t-) alkyne functionalities via a Huisgen reaction. Changes in azide/alkylene molar ratio influence the network structures of the GAP/PPET composites, directly affecting the mechanical properties of the composites. Such characteristics as: crosslink density, tensile strength, Young’s modulus, and breaking elongation showed a similar parabolic dependence on the ratio of azide versus alkylene, where an initial increase was followed by noticeable decline, which strongly depended upon the participation extent of azide/alkyne reaction into the network construction or the hanging degree of PPET chains on the network. At the same molar azide/alkyne ratio, the GAP/t-PPET composites containing higher alkyne-functionality t-PPET showed higher crosslink density and better mechanical properties than those composites that contained two-alkyne-functionality p-PPET. The most favourable values of tensile strength (1.38 MPa), Young’s modulus (4.07 MPa) and breaking elongation (122.5%) were recorded for an azide/alkyne molar ratio of 3:1. Conversely, composites with two alkyne functionalities showed lower glass transition temperatures than composites with three alkyne functionalities, ranging from −79.2 to −75.1 °C (GAP/p-PPET) and from −76.3 to −69.4 °C (GAP/t-PET) respectively.

Hafner et al. [43], in turn, copolymerised epichlorohydrin with 1,2-epoxyhexane, prior to azidating the obtained copolymer to transform the epichlorohydrin repeat units into glycidyl azide units. Although this modification lowered the glass transition point of the copolymer in relation to pure GAP (by 8 ÷ 12 °C), it simultaneously made the copolymers much less energetic than pure GAP, with the heats of decomposition ranging from 1663 J/g to 1821 J/g, compared with 2430 J/g for pure GAP. Expectedly, the copolymer was shown to be less sensitive than pure GAP, with the two polymers showing impact sensitivities of 30 J and 7.9 J respectively and both polymers being insensitive to friction (sensitivity >360 N).

Another interesting approach at producing GAP copolymers was reported by Kim et al. [44], who used a mixture of sodium azide and sodium carboxylate for transforming polyepichlorohydrin, instead of performing azidation solely with the use of sodium azide. The reported method is extremely straightforward and can be easily tailored, by adjusting the sodium azide/carboxylate ratio (resulting in different repeat unit contents) or by using different carboxylates to produce a variety of repeat units. Interestingly, the repeat unit contents differ only slightly from the azide/carboxylate ratio used in the reaction mixture, apparently regardless of the length of the carboxylic acid alkyl chain. Studies of the sensitivity of the copolymers have, expectedly, shown that both GAP and the copolymers are insensitive to both friction and impacts. Although the heats of combustion of the copolymers are larger (−2976 kJ/mol for a copolymer containing 20% of decanoate repeat units) than that of GAP (−2029 kJ/mol), the copolymers contain relatively more carbon and hydrogen atoms than GAP, likely making their oxygen balance values more negative than GAP.

Recently, Filippi et al. reported the synthesis of block copolymers of GAP [45], prepared by using chain extenders (hexamethylene diisocyanate and adipoyl chloride). This approach, however, proved to be problematic, as not only did isolating the desired product prove to be a challenge, but also multi-block by-products were found in the post-reaction mixture, adversely affecting the attainable yield of the product. As such, the authors sought to develop a synthetic procedure affording better control over the obtained products. Their most recent report [46] shows that the mesylate group, mentioned in the previous section, can be introduced not only into the side chains of GAP, as an intermediate for the azidation stage, but also into the terminal hydroxyl groups of GAP, yielding the respective esters. The mesylate esters of GAP and of HTPB can be then subjected to the reaction with HTPB and GAP respectively to produce different types of block copolymers.

A novel concept in the field is the introduction of fluorinated segments as means of improving the mechanical properties of GAP derivatives, well-exemplified by Xu et al. [47], who reported their successful synthesis and investigation of poly(glycidyl azide-co-butane-1,4-diol-co-1,1,1- trifluoro-2,3-epoxypropane). The inclusion of butane-1,4-diol, which the authors used as a reaction activator, is an interesting feature of the synthetic pathway, particularly in light of epoxy-derivative copolymerisation procedures, whose activators are not included in the structure of the copolymer [48]. As such, this choice of procedure may be dictated by the high stability of fluorinated epoxy derivatives or by the beneficial effect of butane-1,4-diol segments on the mechanical properties of GAP-based energetic thermoplastic elastomers, the latter of which was demonstrated by Zhang et al. [49], using it as a chain extending agent for GAP-based energetic thermoplastic elastomers (ETPEs). The obtained fluorine-bearing copolymer was subjected to curing, using Desmodur N100 (**CI2**), a standard isocyanate-bearing agent. The cured system shows a slightly lower glass transition temperature than for GAP (−47.8 °C for the copolymer and −45.3 °C for GAP). The stress-strain curves of the copolymer were similar to those of GAP, showing a slightly higher tensile strength and a noticeably higher elongation at break than in the case of GAP. The authors claim that this copolymer is compatible with a number of materials (HMX, RDX, Al); however, these results are not included in the manuscript.

**Figure 7:**
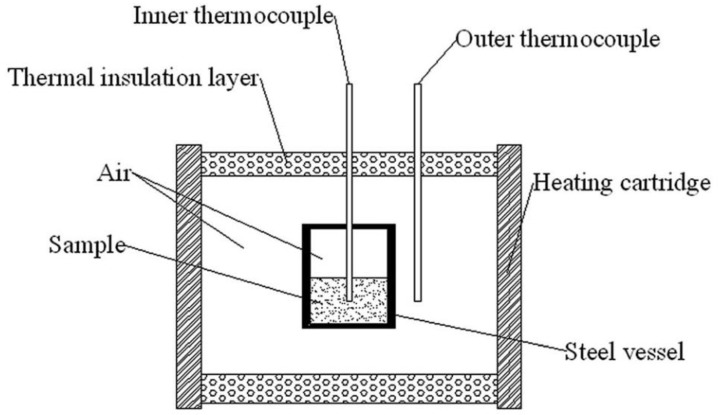
Experimental setup for performing the copolymer/Al mixture cook-off tests. Reproduced from [50] with permission from the Royal Society of Chemistry.

Further work by the authors [50] has led to a slightly altered copolymer structure—instead of attaching a trifluoromethyl group directly to the polyether chain, the strongly electronegative fluorine atoms were introduced in the form of a 2,2,2-trifluoroethoxymethyl group, distancing them from the main polymer chain. The reason for such a modification is not given in the manuscript; however, the polyurethanes based on the modified copolymer exhibit mechanical properties far more favourable than those based on the original fluorinated GAP derivative [47], making the motive readily apparent. Despite achieving only a minor improvement in regards to the aforementioned copolymer, the modification lowers the glass transition of the copolymer in regards to GAP even further, to −49.5 °C. Another interesting feature of this report is a cook-off test (Figure 7), performed for a mixture of the copolymer with aluminium, resulting in a reaction heat value 50% higher than the value for corresponding GAP/Al mixtures.

Continuing their earlier work on ETPEs [49], Wang et al. have recently investigated a series of ETPEs, containing different amounts of hard segments, focusing on their rheological properties [51]. That said, although the authors were able to successfully influence the formation of carbonyl hydrogen bonds and microphase separation transitions by varying the hard segment content, the compounds are yet to be evaluated as energetic binders.

Recently, the authors have also investigated the effect of nitrocellulose on the properties of GAP-based ETPEs [52]. Although they detail an interesting concept, the results of the study appear to imply that the best properties would be achieved for either pure ETPEs/NC or for mixtures consisting primarily (on the order of 90% content) of either one of the components. This is supported by the explosion heat of the ETPE/NC system increasing monotonically with increasing NC content, as well as by the fact that increasing the NC content beyond 10% leads to a dramatic decline in the breaking point elongation for the mixtures, with the expected corresponding increase in tensile strength occurring at NC contents of 40% and above, apart from a minor tensile strength peak at 10% NC, implying some benefits of “doping” ETPEs with NC.

Another type of GAP-bearing energetic thermoplastic elastomer was prepared by Li et al. [53], who copolymerised isocyanate-terminated GAP and poly(3,3-bisazidomethyl oxetane) (PBAMO) prepolymers using butane-1,4-diol linkers. Similarly to the abovementioned study, the Authors utilise isocyanate-bearing reagents despite their drawbacks, showing a lack of viable synthetic alternatives and indicating a potential for improvement. Unlike Wang’s report, however, a difunctional isocyanate is used, yielding a linear copolymer rather than a cross-linked one. In this report, PBAMO is used as the hard segment and GAP as the soft segment. In their experiments, the Authors maintain a 1:1 weight ratio of the two prepolymers, focusing on investigating the effects of diluting them intra-molecularly with the butanediol segments, while increasing the content of hard carbamate segments produced in the reaction between the isocyanate and hydroxyl groups. This increase expectedly translates into increasing the stiffness of the material, with the best mechanical properties being found for a system containing 30% of the isocyanate and butanediol. Simultaneously, an increase in the glass transition temperature with increasing the content of hard segments is noted.

A different approach to preparing GAP-based elastomers was reported by Deng et al. [54], who blended GAP with other polymers prior to curing rather than using GAP-containing copolymers. The authors supplemented GAP with additions of up to 30% (*w*/*w*) of poly (ethylene oxide-co-tetrahydrofuran) (PEOTHF) and polyalkylene oxide (PAO). Such polymer mixtures were then transformed into copolyurethane elastomers via the use of isocyanate curing agents (**CI1**, **CI2**). These copolyurethanes were found to exhibit mechanical properties significantly improved in relation to those of polyurethanes made from pristine GAP. Although copolymers prepared using GAP-PEOTHF mixtures showed improved properties, copolymers based on GAP-PAO mixtures showed even more superior properties. Interestingly, for systems containing more than 15% PAO, the regularity of the polymer begins having an effect, as the authors observed the crystallisation of PAO segments in the copolyurethanes, leading to further improvements of the mechanical properties of the systems.

The concept of blending GAP and poly(ethylene oxide-co-tetrahydrofuran) was further investigated by Li et al. [55], in hopes of exploiting the favourable low temperature properties of PEOTHF. The Authors prepared a series of such blends and cured them using a mixed isocyanate system, composed of Desmodur N100 (**CI2**) and toluene diisocyanate (no information is given on which of the commercially available isomers/isomer mixtures was used, with the choice potentially affecting the properties of the cured systems to a significant extent). An interesting feature of this study is the investigation of the miscibility of the two polymers prior to curing, revealing that the binary system exhibits a lower critical solution temperature at approx. 30 °C—an extremely important parameter in terms of potential industrial-scale production and processing of such a binder system. Interestingly, this limited miscibility does not translate to any issues in a cured system, as found in the course of the authors’ extensive exploration, with a 1:1 ratio of GAP to PEOTHF yielding the best mechanical properties. These properties can be easily tailored by varying the GAP/PEOTHF ratio, the hydroxyl/isocyanate ratio and the composition of the curing system (Desmodur N100/toluene diisocyanate ratio), as explored and detailed in the report by the Authors. Interestingly, even when the manuscript indicates that the same polyurethane was investigated (NCO/OH, GAP/PEOTHF and CI2/toluene diisocyanate ratios being identical), different mechanical properties are reported, implying that the average deviation in experimental values should be determined.

The authors continue investigating the properties of GAP/hydroxyl-terminated poly(ethylene oxide-co-tetrahydrofuran (GAP/PEOTHF) copolyurethane networks, reporting on such polymer networks produced via a stepwise curing process [56] rather than via a conventional one-step process, as in their earlier report, mentioned above. The authors investigated the effects of both cross-linking density (as expressed via the hydroxyl/isocyanate molar ratio) and GAP content on the mechanical properties of copolyurethanes produced in accordance with the two-stage (stepwise) and one-stage curing procedures. Interestingly, the stepwise curing procedure yields improved mechanical properties for low-GAP (below 50% content) copolyurethanes, while yielding a slight deterioration in the properties of high-GAP systems. Again, as in the abovementioned work, an issue in terms of results’ repeatability is encountered. This issue is exemplified by a system, with GAP/PEOTHF and NCO/OH ratios equal to 50:50 and 1.3:1 respectively, having shown slightly different mechanical properties, depending on whether the former or latter ratio was being varied in the experiments.

Instead of modifying GAP itself, in order to develop an energetic binder, Pei et al. [57] investigated a GAP/3,3-bis(azidomethyl)oxetane (BAMO) copolymer, which was supplemented with copper(II) oxide nanoparticles, acting as a combustion modifier. The use of metal oxides as combustion modifiers is well-known and, although nano-CuO is reported to have been used alongside AP and RDX, its effect on the decomposition of energetic binders has been explored in less detail. Thermal studies of the CuO-doped and pristine GAP-BAMO copolymers revealed that the nanoparticles lower the first stage decomposition temperature of the binder by more than thirty degrees, while increasing the heat of decomposition by even 12% and lowering the activation energy from 158.7 kJ/mol for the pristine copolymer to 147.5 kJ/mol for a copolymer containing 50% (*w*/*w*) CuO. The authors utilised mass spectrometry to investigate the changes in the mechanism of the binder decomposition reaction, postulating that CuO promoted the breakage of N-N bonds in the azide groups, leading to a more complete release of nitrogen during decomposition of the binder sample.

Bellan et al. [58] adopted an approach similar to the abovementioned; rather than using GAP, however, the authors prepared polyurethanes using 2,2-bis(azidomethyl)propane-1,3-diol (BAMP) and 2,2-dinitropropane-1,3-diol (DNPD). The properties of the resultant compounds were determined experimentally and theoretically, with both systems showing no sensitivity to friction and lower sensitivity to impact than GAP. The reaction products have relatively low molecular weights, corresponding to polymer chains no longer than ten repeat units and as such may be considered oligomers. As such, by adjusting the molecular weight of these compounds, their properties may be tuned, potentially allowing subsequent improvements to their sensitivity to impact and electrostatic discharge.

Chizari and Bayat [59], in turn, attempted to improve the mechanical properties and low-temperature performance of GAP by equipping it with terminal polycaprolactone blocks. An interesting feature of their work is the use of GAP with relatively low molecular weight (M_N_ = 1006 g/mol), yielding a triblock copolymer (polycaprolactone-GAP-polycaprolactone) with M_N_ of only 1794 g/mol. The glass transition temperatures found for this low-MW GAP and its triblock copolymer were respectively −48.8 °C and −64.3 °C, far more favourable in the case of the copolymer.

Hafner et al. [60] report an attempt to modify the thermal and mechanical properties of GAP and poly(3-nitrato-methyl-3-methyloxetan) (poly(NIMMO)) through the use of the dinitro and bisazidomethyl derivatives of 1,3-propanediol as energetic plasticisers. All investigated plasticisers exhibit glass transition temperatures below −70 °C and decomposition temperatures above +230 °C. The plasticiser compounds are volatile, with initial weight losses being reported at temperatures in the range of +70 ÷ 100 °C, comparable with +80 °C for N-butylnitratoethyl nitramine (Bu-NENA) under the same conditions. It is worth mentioning that the authors took care to investigate the sensitivity of the pristine plasticisers, finding them insensitive to impact (>40 J) and friction (>360 N), more favourable than Bu-NENA, whose friction sensitivity is cited to be 108 N. Studies of the plasticising ability of the compounds towards GAP and poly(NIMMO) revealed that both the dinitro and bisazidomethyl derivatives perform similarly to Bu-NENA in mixtures with GAP, both at low and elevated temperatures. In the case of poly(NIMMO), the dinitro derivatives were again comparable with Bu-NENA, while the bisazidomethyl derivatives showed noticeably lower performance (viscosity of the plasticiser/polymer mixture being roughly 50% higher than in the case of Bu-NENA). Unfortunately, the authors did not evaluate the effects of their compounds on the properties of cured GAP/poly(NIMMO). As such, the synthesised compounds are surely promising thinners but might not perform as favourably as plasticisers.

A different approach to reactive energetic plasticisers for GAP is reported by Bodaghi and Shahidzadeh [61], who opted for using polymeric plasticisers. The authors prepared a series of propargyl-terminated poly (glycidyl nitrate) derivatives, able to react with GAP via the azide-alkyne reaction, resulting in a GAP-graft-poly(glycidyl nitrate) system. Interestingly, systems containing 5% and 10% grafts were readily plasticised, as shown by the decreased glass transition temperature, respectively by 6.1 K and 4.6 K. Conversely, the copolymer containing 15% grafts showed a glass transition temperature higher than that of GAP, clearly indicating that the content of poly (glycidyl nitrate) grafts needs to be carefully optimised.

**Figure 8:**
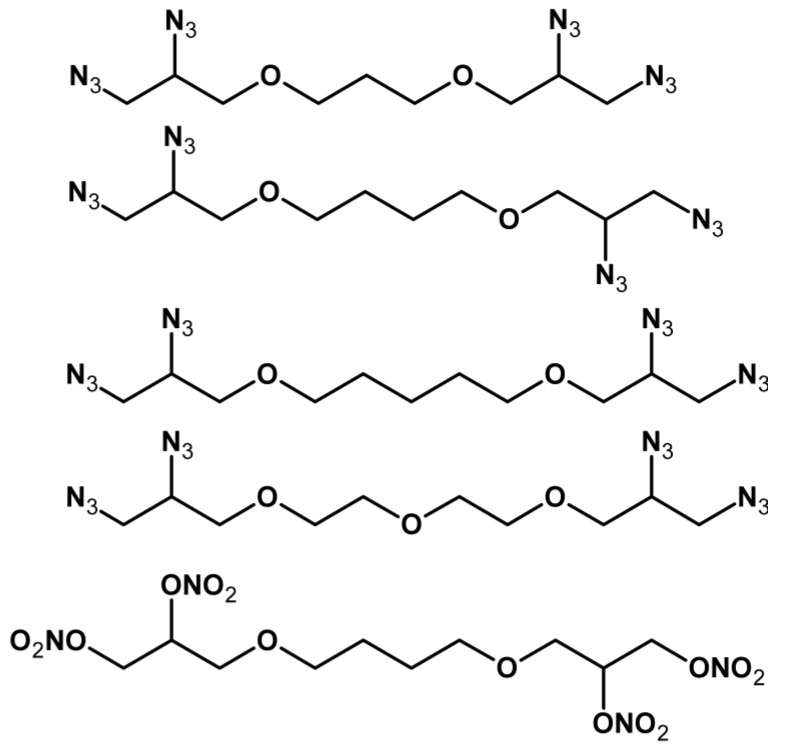
Azide-bearing oligoether derivatives tested as plasticizers for GAP by Baghersad et al. Reference plasticizer: glycidyl nitrate dimer (lowest). Based on structures shown in [62].

The concept of plasticising GAP was also investigated by Baghersad et al. [62], who focused on a series of dimeric and trimeric ethers, whose end groups were each functionalised with two azido groups (Figure 8). For all of the plasticisers, their addition either to GAP or polyurethane prepared from GAP resulted in a significant decrease in the glass transition temperature of the system. Thermochemical experiments were conducted, confirming the expected high heat release capability of each compound; at the same time, the compounds were found to be insensitive to friction, up to a 360 N stimulus, using a standard BAM apparatus. Although no information is given about their impact sensitivity, the investigated compounds appear promising for use in the development of GAP-based propellants and, potentially, advanced explosives.

Although ionic liquid polymers, derived from GAP via the azide-alkyne reaction, have been reported in literature [63], Fareghi-Alamdari et al. [64], have been the first to employ the ionic moieties as plasticisers for GAP. The authors employed bromide and dicyanamide salts of 1-methyl-3-propargyl imidazolium as the reactive ionic plasticisers, with the dicyanamide salt also fulfilling the part of an energetic plasticiser. Interestingly, while both salts significantly reduce the glass transition temperature of GAP (reported as −38 °C), GAP containing 40% grafts of the dicyanamide salt showed the lowest glass transition temperature of approx. −60 °C, as well as noticeably lowered viscosity, unlike GAP with grafts of the bromide salt, which increased viscosity, regardless of the grafting density. A considerable drawback of this modification, however, is that grafting lowered the heat of combustion of the modified GAP by 6 ÷ 22%, in comparison with the heat of combustion of the unmodified GAP, depending on the type of salt and grafting density.

The same group also studied the reaction of GAP with other energetic plasticizers, bis-(2-ethylhexyl)-but-2-ynedioate (BEHB) and bis(2-nitropropyl)-but-2-ynedioate (BNPB) [65]. Covalently linked reactive plasticizers BEHB and BNPB effectively reduced the viscosity of GAP from 5.5 Pa·s to 2.5 and 1.5 Pa·s. GAP/Reactive plasticizers (60/40, *w*/*w*) showed a reduction in glass transition temperature (up to −47.7 °C in case of GAP/BNPB), as well as decrease in heat of combustion (24.52 kJ/g for pure GAP, 17.31 kJ/g for GAP/BEHB and 21.42 kJ/g for GAP/BNPB).

Zhao et al. [66] took a more basic approach and investigated the compatibility and interactions of GAP with N-butyl-N-(2-nitroxy-ethyl)nitramine (Bu-NENA) and bis(2,2-dinitropropyl)- formal/acetal (BDNPF/A), both used as insensitive energetic plasticizers, in a combined theoretical and experimental study. The authors found that both plasticizers exhibit strong intermolecular interactions with GAP and both are good solvents for the polymer; Bu-NENA, however, was found to be the superior plasticizer for GAP, as shown by both calculations and experiments.

Another theoretical study in this subject was conducted by Yang et al. [67], who investigated 2-azidoethyl nitrate ester (AENE) as a potential plasticiser for GAP and nitrocellulose that could replace nitroglycerine in this regard. Utilising density functional theory calculations, the authors predict that AENE will show good compatibility with both GAP and NC, while providing superior mechanical properties to its composites than NG, making the compound an interesting candidate for experimental evaluation.

The compatibility of GAP and DNDA-5 (2,4-dinitro-2,4-diazapentane) was also investigated, using thermal analysis [68]. For instance, in case of GAP/DNDA-5 one observed lowering of glass transition temperature by 1.9 °C when compared to the pure binder. Interestingly, density functional theory (DFT) method predictions did not support the results of thermal studies, because they indicated positive free Gibbs energy of GAP–DNDA-5 interaction (3.3 kJ/mol).

Another approach was presented in [69], where synchronous dual curing system was used to fabricate glycidyl azide polymer–hydroxyl terminated polyether semi-interpenetrating polymer network (GAP–HTPE semi-INP). Semi-IPN allows combining both linear and cross-linked networks with stable phase and enhanced properties (e.g., tensile strength, breaking elongation, thermal stability) due to its unique structure. By ensuring the synchronisation of catalytic urethane reaction of HTPE/isocyanate and azide–alkyne click chemistry of GAP/dimethyl dipropargylmalonate, a homogenous GAP–HTPE semi-IPN was fabricated, which exhibited tensile strength of 5.72 MPa and 194% elongation at break. Analysis of thermal decomposition revealed relatively good thermal stability of the analysed material (nonisothermal differential scanning calorimetry– thermogravimetry–pyrolysis mass spectrometry coupling analysis (DSC–TG–PyMS) showed exothermic peak temperatures at 235 °C and 412 °C corresponding to the pyrolysis of GAP and attributed to the depolymerisation and pyrolysis of HTPE, respectively) with remarkable reduction of azide ion gaseous product comparing with GAP based polyurethane.

### 2.4. Chemical Transformation of the Azide Groups of GAP

An altogether different approach to modifying the properties of GAP is to transform the azide functional groups of the polymer into other functionalities. Although this commonly involves a methodology similar to that employed when using alkyne-bearing species to cure GAP, other reactions are also utilised (e.g., for converting the azide groups into nitramine groups).

The key difference between the former type of transformation and curing is that instead of using a difunctional alkyne, a monofunctional alkyne is used (as per the reaction presented in Scheme 3a and opposed to the reaction used for curing, shown in Scheme 3b). Consequently, instead of connecting the GAP chains with each other, the alkyne connects the desired moiety to one GAP chain, thereby making it possible to introduce various substituents into the short “side-chains” of GAP.

**Scheme 4:**
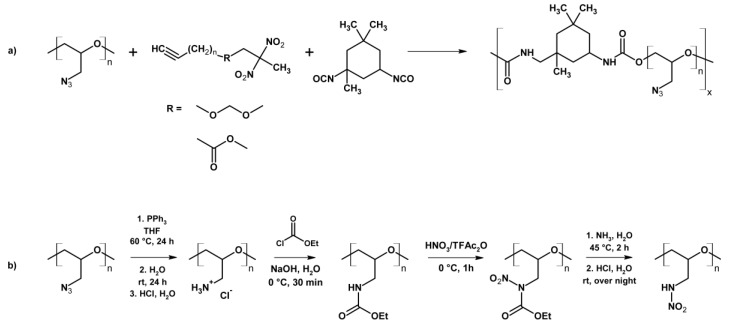
Reaction schemes of reported chemical transformations of GAP azide groups.

This type of GAP transformation is well-exemplified by the report by Ma et al. [70]. The Authors simultaneously cured GAP with a difunctional isocyanate curing agent (reaction through hydroxyl end groups) and, by using a reactive energetic plasticizer, converted some of the azide functional groups into triazole linking moieties, equipped with a dinitroalkyl functionality (Scheme 4a). The authors thoroughly investigated the chemical identity of the attainable reaction products and their properties, reporting improvements in term of the viscosity of the GAP prepolymer and mechanical performance of the resultant energetic polyurethanes. Although these systems appear potentially promising, their energetic properties and sensitivity to stimuli are yet to be uncovered.

Rather than using the azide group as a template for a moiety linking GAP with the desired substituents, the azide group can also be transformed directly, i.e., through its reduction to an amine group. Such an approach was taken by Betzler et al. [71], who utilised it to prepare a glycidyl nitramine polymer (GNAP). GNAP is produced from GAP through a four-stage synthetic pathway, involving steps to protect the incipient amine groups from being nitrated more than once (Scheme 4b). The final polymer is solid, as opposed to the liquid GAP. On the one hand, as the authors point out, this reduces the need for curing agents, on the other hand, it can also lead to issues with achieving sufficient homogeneity of GNAP-based compositions. IR spectroscopy is used to monitor the individual transformations, with the spectrum of the final polymer containing both the nitramine-originating signals and N-H stretching band, which is consistent with the expected structure of the product but does not exclude free amino groups being present in the polymer; establishing the degree of amine/nitramine conversion would most likely require an in-depth high resolution NMR spectroscopic investigation. Although GNAP shows a slightly lowered decomposition temperature (approx. 170 °C, with 216 °C being reported for GAP), it is far less sensitive to impact than GAP (40 J, in comparison with 8 J for GAP) and, much like GAP, is insensitive to friction (>360 N).

### 2.5. Properties of GAP, Cured GAP and its Derivatives

Due to the properties of GAP being well-known, reports about the properties of GAP and its derivatives are relatively less common than other works, much as is the case with reports concerning advances in the synthesis of the polymer. One particular trend, emerging among such works, is that of creating theoretical models to predict the properties of GAP and its derivatives, as well as their performance in various formulations. This trend manifests both as theoretical compatibility studies and as more in-depth works, such as that of Khan et al. [72]. In that work, the authors calculated the enthalpies of formation and band gaps for HTPB, GAP and, subsequently, azido-HTPB, a novel potential energetic binder. An interesting aspect of this work is the postulated correlation between the breadth of the band gap and the sensitivity of the materials to stimuli.

Another in-depth exploration, focusing on developing a model of the mechanical properties of GAP was conducted by Ma et al. [73]. The authors investigated the tensile strength of the polymer as a function of both temperature and strain rate, subsequently utilising the data to derive a model accounting both for the strain rate effects and for the softening of GAP with increasing temperature.

It was mentioned before that GAP itself is often exchanged for one of its derivatives, as those next-generation materials, whose structure allows the relevant deficiencies of GAP to be countermanded and additional, desirable properties to be bestowed, are tailored for particular applications. In line with this approach, Pei et al. [74] investigated a copolymer of GAP with poly(3,3’-bis(azidomethyl)oxetane) (BAMO), in terms of its compatibility with commonly used components of pyrotechnic compositions—oxidising agents, plasticizers and fuels. The compatibility is assessed using thermal properties, with the difference between the peak maximum temperature of the pristine systems and the mixed systems being used as the criterion. Interestingly, in the case of most investigated systems, there is little interaction between GAP-BAMO and the other component, with the differences in peak temperatures being marginal. In one case, however, i.e., a mixture with DNTF, the use of GAP-BAMO is not advisable, as the mixture is significantly more prone to decomposition than either of its components.

Based on both theoretical considerations and the in-depth investigations of the properties of GAP, the issue of detecting GAP (and other azide-bearing species) is both interesting in terms of research and important in terms of public security. This branch of research is exemplified by the work by He et al. [75], who developed a colorimetric sensor for this purpose, utilising the reaction between the azide groups of GAP and alkynes (as per the general mechanism depicted in Scheme 3). The authors utilised gold nanoparticles as chromophores instead of the traditional organic chromophores, due to more favourable (significantly higher) extinction coefficients; a frequent approach in the development of sensors for detecting other explosives [76,77,78]. The reported limit of detection for GAP was found to be 0.1 μg/mL, utilising the naked eye to evaluate the colour change. The selectivity of the sensor was tested against several other common macromolecular species: ethylene-vinyl acetate copolymer, poly (ethylene oxide)-poly(propylene oxide)-poly(ethylene oxide) triblock copolymer, polyvinyl pyrrolidone, phenol formaldehyde resin, carboxymethyl cellulose, polyvinyl alcohol, and gelatine; the results reveal a very strong response for GAP and only marginal for the other, interfering, species. Unfortunately, the authors did not explore the possibility of detecting other azide-bearing polymers, such as PBAMO, poly(NIMMO) or PAMMO, using this sensor; it would be expected that the sensor is selective against azide groups rather than being specific only for GAP, making it even more versatile than reported.

**Figure 9:**
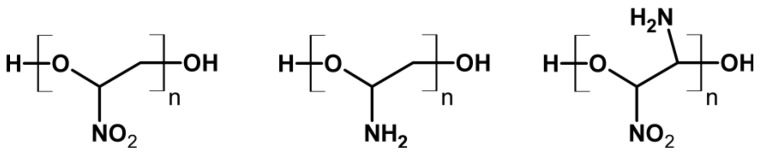
GAP derivatives studied by Lu et al. Based on structures shown in [79].

Lu et al. [79], in turn focused their efforts at utilising molecular dynamics theory to predict the glass transition temperatures and mechanical properties of GAP and three of its derivatives (Figure 9). The physicochemical property values predicted for GAP were in good agreement with experimentally determined values, implying that the adopted theoretical model is adequate and that the predictions for the derivatives will also show good accuracy.

## 3. Applications as a Component of Solid Propellants

The primary role of GAP in solid propellant formulations is that of an energetic binder, improving or bestowing favourable mechanical properties upon the propellant, while affecting the combustion parameters of the propellant only to a minimal extent. When considering this issue, it is worth noting that even inert (non-energetic) binders do not directly affect the combustion rate of the relevant formulations; this was known as early as 1972, with a report by Cohen and Fleming [1] stating: *“In practice, the inert binders used in production composite propellants over the past 15 years have only secondary effects on burning rate. At a time when propellant technology limited oxidizer particle size selectability, total solids, and use of additives, this secondary effect was sufficient to base binder selection on burning rate in some instances (other factors being about equal)”*. Rather than affecting the combustion rate of the formulation, the choice of binder has a significant effect on the net combustion heat of that formulation, either by being inert and acting as “thermal ballast” or by undergoing endothermic decomposition.

Another parameter strongly affected by the choice of binder for a particular formulation is its sensitivity to initiating stimuli. This dependence originates both from the sensitivity of the binder itself, as mentioned in the introduction, and from the ability of the binder to interact with and “stabilise” the components of the formulation. The latter can be achieved by a number of mechanisms, i.e., a polymeric binder can act as a micro-scale structural support, dispersing the energy of mechanical stimuli and lowering the sensitivity of a formulation, but it can also be applied as a coating to the grains of a substance exhibiting high sensitivity to mechanical stimuli and act as a “cushion”.

In the case of solid propellants, whose manufacture requires the curing of a polymeric binder (commonly referred to as cast-cured composite propellants), such as is the case for GAP-based formulations, the viscosity bestowed upon the formulation by the not-yet-cured binder is an important factor to take into account, strongly affecting the processing of such a formulation. Although a lower viscosity of the formulation is generally desirable in terms of processing, an excessively low viscosity may promote sedimentation of the formulation suspension during binder curing; as such, formulation viscosity should be taken into consideration alongside binder curing kinetics and the sedimentation profile of the propellant formulation suspension. The compromise between viscosity and curing time can be approached both practically and theoretically, as in the case of Xu et al. [80], who investigated GAP as a binder for aluminium nanoparticles (nano-Al), alongside poly(ethylene oxide-co-tetrahydrofuran) (PEO-THF) and HTPB. The authors focused on the rheological properties of the suspensions of nano-Al with each of the three binders, studying them at several temperatures and determining the flow activation energy for each system, reporting the highest value (52.07 kJ/mol) for nano-Al/GAP and the lowest (1.506 kJ/mol) for nano-Al/ PEO-THF.

Aside from reports dedicated to the applications of formulations involving GAP and its derivatives as binders and practical handling of the abovementioned issues, many works are dedicated to more sophisticated subjects. One such case is an attempt to provide an alternative to an existing fuel, carbon black. Although significantly more expensive, C_60_ fullerene can be used as a replacement for carbon black in rocket fuels, increasing the combustion rate and lowering the NO_x_ content in the post-combustion gasses [81,82]. C_60_ can be enhanced by introducing energetic substituents, such as the nitro group, onto it; the stability of such derivatives is, however, an issue. Huang et al. [83] report a novel method for circumventing this stability issue—rather than grafting energetic groups directly onto the fullerene, the authors functionalise the fullerene with GAP and, indirectly, with azide groups. The authors synthesise GAP-functionalised fullerene by using a bromomalonic GAP ester intermediate, reporting a yield of 83%. An extensive suite of instrumental techniques is used to confirm the structural identity of the obtained GAP-functionalised fullerene, with thermal analysis being employed to investigate the thermal decomposition of the compound. An interesting feature of the report is the use of thermogravimetric analysis coupled with infrared spectroscopy, allowing the authors to investigate the chemical nature of the species existing in the sample during its thermal decomposition and to suggest a potential reaction pathway for this process.

### 3.1. Double-Base Propellants

A relatively straightforward “double base” propellant formulation, based on GAP (used as a replacement for nitroglycerine) and NC was reported by Zhao et al. [84]. The propellant is fabricated by swelling NC in acetone prior to the addition of GAP; this is followed by slow evaporation of the solvent in moulds, allowing the propellant to take the desired shape while drying, with the pre-dried, shaped samples being subsequently vacuum-dried to produce the final product. Different GAP/NC ratios were investigated, as well as a reference 40% NG double base propellant. Although 30% GAP was insufficient for plasticising NC, as shown by SEM investigation, propellants containing 40% and 50% GAP achieved plasticisation and good structural homogeneity; the latter being also confirmed by tensile strength measurements (very similar results were obtained for different batches of a propellant containing the given amount of GAP). The thermal properties of the composite, on the other hand, were largely unaffected by the introduction of GAP, with the temperature of the decomposition onset being similar to that of pristine NC.

**Figure 10:**
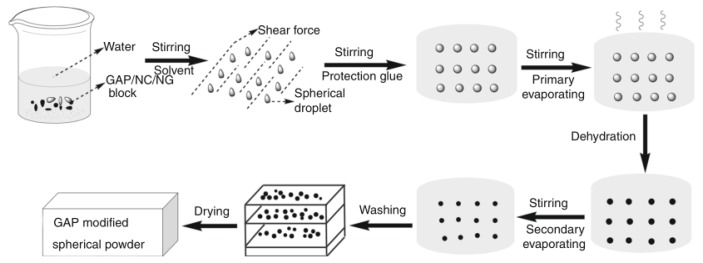
Step-by-step scheme of the procedure for preparing GAP-modified NC/NG spherical powder [85].

Continuing their earlier work [33], Wu et al. report a novel GAP-modified double base powder formulation [85]. GAP is introduced as a modifier into the nitrocellulose (NC)/nitroglycerine (NG) block by means of a multi-step process (Figure 10), yielding a spherical powder, which is then used as a component in a propellant formulation (7.5 ÷ 13.0% content), whose exact composition is given by the authors. 

The pristine GAP-modified powder was found to be spherical for GAP contents below 30%, with lower GAP contents yielding smoother spherical structures. Thermal studies show four decomposition stages, corresponding to the two stages observed for non-modified double base powders and the two stages observed for pristine GAP, with the modified-powder showing slightly different onset and peak maximum temperatures. 

**Figure 11:**
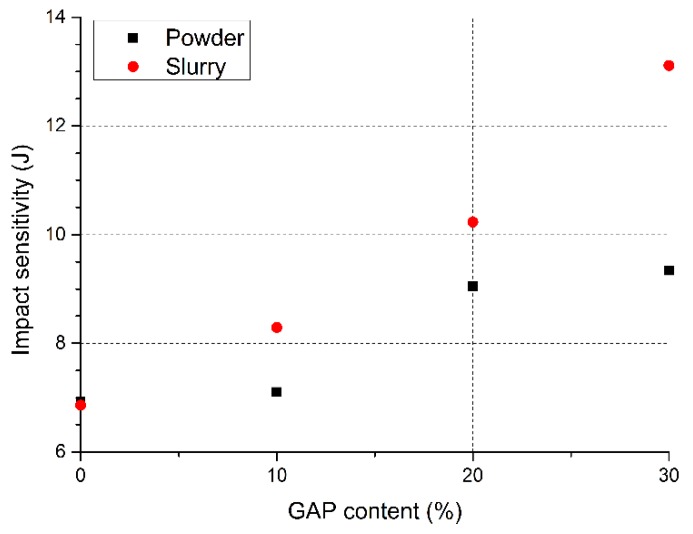
Impact sensitivity of GAP-based double base powder and propellant formulation as a function of GAP content. Based on data in [85].

This behaviour implies little interaction between the components, with the difference in thermogravimetric profiles being more akin to a weighted average of the profiles of the two parent systems than a new quality by itself. The true significance of the GAP modifier, however, is revealed in the impact sensitivity experiments, with both the powder and slurry containing 30% GAP being roughly half as sensitive as the non-modified systems (Figure 11). The authors later revisited the subject [86], focusing on investigating the more fundamental properties of GAP-modified double base powders and their decomposition mechanisms and products, proposing a thermal decomposition mechanism for the propellant system.

### 3.2. Composite Propellants

**Scheme 5:**
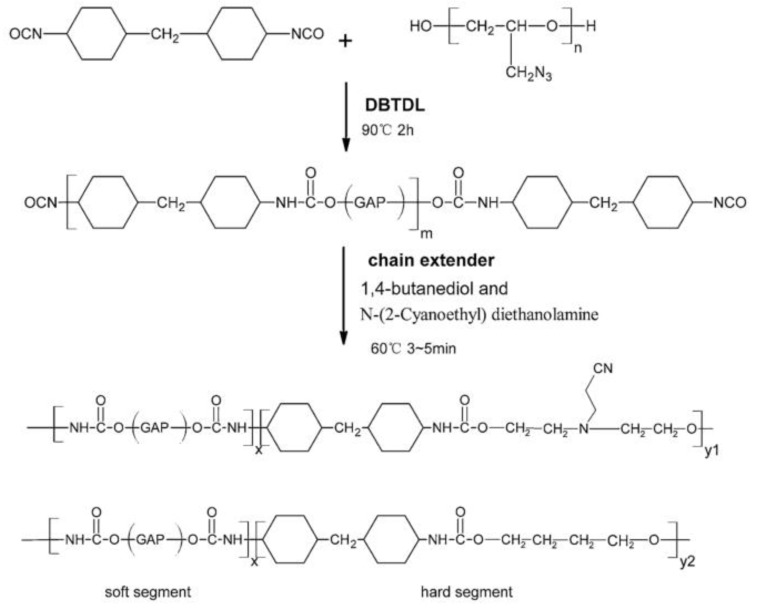
Synthetic pathway to the preparation of GAP-based ETPEs [49].

Many binders commonly used in the manufacture of solid propellants are thermoplastic elastomers that have been cross-linked using isocyanate-bearing agents. Not only are such binders non-energetic, detrimentally affecting the attainable performance of pyrotechnic compositions, but the isocyanate cross-linking agents are toxic, environmentally harmful and often offer insufficient stability due to their reactivity. To alleviate some of these drawbacks, Zhang et al. [49] proposed the use of GAP-based energetic thermoplastic elastomers (ETPEs). The soft GAP segments were supplemented with hard segments, prepared through chain extension (Scheme 5), using butane-1,4-diol and N-(2-cyanoethyl) diethanolamine, linked with the GAP segments through an interesting isocyanate curing agent, methylene bis(4-cyclohexylisocyanate) (**CI3**) rather than the more common hexamethylene diisocyanate (**CI4**). The properties of the ETPEs were controlled by the content of the N-(2-cyanoethyl) diethanolamine in the chain extension mixture and a wide range of concentrations was investigated. The obtained series of ETPEs were then used to prepare RDX-based propellants, in order to test their feasibility for application as binders. An interesting highlight of the article is the authors’ in-depth analysis of the IR spectra of the ETPEs, giving an insight into the ordering of the polymer chains and their interactions through hydrogen bonds. The ETPEs themselves were found to be insensitive to friction (>360 N) and impact (>40 J) and, interestingly, showed the best performance, in terms of mechanical properties and resistance to dewetting, for a 1:1 N-(2-cyanoethyl) diethanolamine to butane-1,4-diol ratio in the chain extension mixture.

Jensen et al. [87] report a thorough theoretical study of the potential of 5-amino-3-nitro-1,2,4-triazole and 3-nitro-1,2,4-triazol-5-one as high explosives and rocket propellants. An interesting conclusion of this work is that GAP would be a promising choice of binder for these systems, predicting that specific impulses above 2600 m/s could be achieved for such a propellant system.

**Figure 12:**
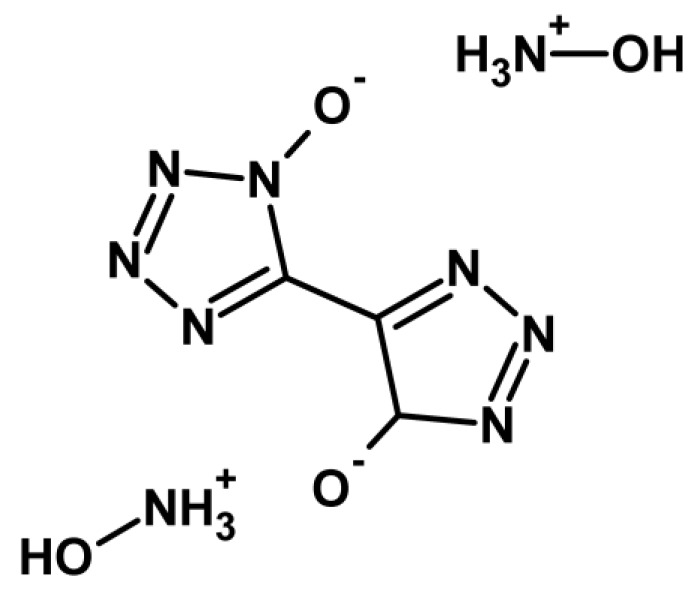
Structural formula of dihydroxylammonium-5,5’-bistetrazole-1,1’-diolate (TKX-50) [90].

The application of GAP in energetic solid propellants for missile weapon systems was considered in conjunction with two different explosive materials: ionic 5,5’-bistetrazole-1,1’-diolate (TKX-50) [88], whose application is relatively novel in the field, and one of most commonly used non-ionic explosive cyclotetramethylenetetranitramine (HMX) [89]. The binding energy between GAP and TKX-50 (Figure 12) is larger than for GAP and HMX (163.35 kcal/mol and 148.98 kcal/mol, respectively), indicating stronger interactions between GAP and the crystalline surface of TKX-50 than between GAP and HMX. This relation is also confirmed by the adhesion energy between GAP and the considered explosives. In case of TKX-50 it is 62.61 mN/m and for GAP/HMX interfaces it equals to 57.51 mN/m. A possible explanation of these results involved the radial distribution function analysis, which indicated existence of stronger hydrogen bonds and van der Waals interactions between interface of GAP molecules and TKX-50. The spreading coefficients for GAP matrix and fillers were both positive, suggesting fine wetting behaviours of GAP molecules on the surfaces of both solid particles. Considering mechanical properties of GAP/HMX and GAP/TKX-50 composites (tensile strength, initial modulus and resistance to tension), it was found that the GAP/TKX-50 exhibited better characteristics than GAP/HMX, which makes GAP/TKX-50 a promising candidate for future applications.

GAP can also be used as a binder for rocket fuels based on magnesium, as shown by Zygmunt et al. [91], who investigated it, alongside HTPB as a binder for Mg/ammonium perchlorate composites. Studies of such rocket fuels showed that, while for HTPB the lowest investigated content of the binder (10% *w*/*w*) yields the highest specific impulse, in the case of GAP, the highest specific impulse is achieved for a high (30%) content of the binder, potentially allowing the mechanical properties of the composite to be improved much further, an issue that the authors are yet to investigate. Expectedly, composites utilising GAP showed higher specific impulses for the same Mg contents than those utilising HTPB.

Nitramines, such as RDX, are often used in solid propellants; in high concentrations, however, can adversely affect the performance of the propellant and reduce the safety of its applications [92,93]. This issue is often resolved by coating the surface of the nitramine with a less sensitive material. Inert coatings are undesirable as they can limit the energy efficiency of the propellant; as such, energetic binders, such as nitrocellulose (NC) are primarily used. Even so, an important drawback of nitrocellulose is its low viscosity, which, when used to directly coat the nitramine, may induce a dehumidifying phenomenon. Consequently, Ye et al. [94] investigated the possibility of supplementing NC with GAP, by preparing RDX/NC/GAP composites via the solvent/non-solvent method. Although the authors found that neither NC nor GAP are capable of influencing the crystal structure of RDX, the coating is not without effect, as a significant drop in impact sensitivity was observed for the coated sample in relation to a comparable pristine RDX sample (H_50_ values of 40.74 cm and 23.40 cm, respectively). Interestingly, DSC studies of pristine RDX and the composite have shown that the two binders have only a limited effect on the thermal decomposition of the nitramine. Comparison of the mechanical performance of solid propellants using either RDX or RDX/NC/GAP as filler showed relatively similar results, with the composite exhibiting the best properties.

A different approach to enhancing the properties of RDX is to offset its strongly negative oxygen balance (−21.6%). A good example of this approach is the work by Li et al. [95], who report a composition consisting of RDX, ammonium perchlorate (oxygen balance of +34%) and GAP. The Authors investigate a number of formulations, altering the RDX/ammonium perchlorate content ratio, in order to study the effects of changing the net oxygen balance of the composition on its performance. Interestingly, thermal studies (DSC) of the composition samples show clearly separated decomposition signals for RDX and ammonium perchlorate, regardless of their content ratio. Such a signal separation (peak separation of roughly 100 K) implies that the combustion rate of the composition will not be stable and will shift from that of RDX initially to that of ammonium perchlorate once most of the composition undergoes combustion.

**Figure 13:**
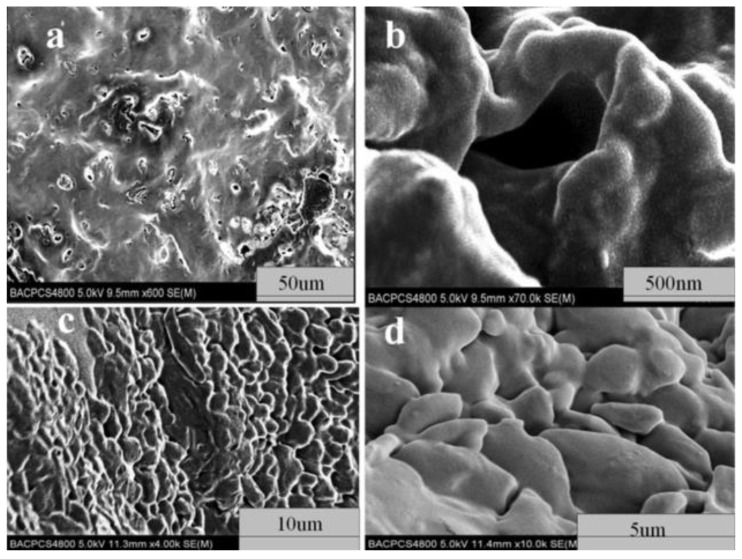
SEM photographs of GAP xerogel (**a**) morphology; (**b**) microscopic pore structure; and of (**c**), (**d**) RDX/GAP composite microstructure [96].

An interesting GAP-based composite was reported by Li et al. [96], who used the sol-gel method to prepare GAP nano-networks, within whose pores another material could be deposited (Figure 13), depending on the intended purpose of the composite. For the purpose of the report, the Authors used RDX as the other component of the composite; the RDX incorporated into the composite was found to exist as the most favourable, in terms of performance, α-RDX crystalline phase. When the quthors varied the ratio of the components, they found that the size of the RDX particles increased with the content of the incorporated RDX, allowing the properties and performance of the composite to be fine-tuned. One highlight of the report is that the composite was less sensitive to impact (h_50_ = 30.2 cm) than not only pristine RDX (12.8 cm) but also less sensitive than a RDX/GAP blend (15.59 cm).

Futko et al. [97] approached a RDX/GAP solid propellant from a theoretical standpoint, opting to model its behaviour and combustion characteristics in a rocket engine, providing much information about the thermal efficiency of such solid propellants in dedicated combustion chambers.

An alternate approach to RDX/GAP composite propellants was adopted by Jensen et al. [98], who supplemented and replaced the RDX in the formulations with 1,1-diamino-2,2-dinitroethylene (DADNE, FOX-7). The Authors investigated several different formulations, altering the content of FOX-7 and its particle size distributions. Interestingly, while the density, oxygen balance and specific impulse of the compositions were relatively constant, regardless of the RDX/FOX-7 ratio, the exhaust velocity was found to be the highest in the most RDX-rich formulation. Similarly, when RDX was wholly replaced with FOX-7, the combustion was found to be erratic in terms of rate, possibly indicating some drawbacks of using FOX-7 in this type of formulations. Unfortunately, while the authors investigated the formulations in much detail, their results have not been standardised against a formulation based solely on RDX, making evaluation of their propellant formulations non-straightforward.

Abd-Elghany et al. [99], in turn focus on employing a new oxidising agent, 2,2,2-trinitroethyl-formate (TNEF), in propellant formulations using GAP. Propellants containing 14% of cross-linkedGAP cured using hexamethylene diisocyanate (**CI4**), and 86% of either TNEF or ammonium dinitramide (ADN) were investigated. Interestingly, propellants based on TNEF showed a significantly higher specific impulse value (250.1 s) than those based on ADN (202.1 s). Similarly, the TNEF-based propellants were also found to be more stable, with their decomposition taking place at 210.1 °C, as compared to 183.5 °C for ADN-based propellants. An interesting point made by the authors is that the TNEF-based propellants are chlorine-free, unlike propellants based on ammonium perchlorate (AP). Although this is a clear advantage, it would be without doubt worth to include AP-based propellants in the study, alongside TNEF- and ADN-based propellants, particularly if TNEF-based propellants could be expected to show more favourable properties than AP-based propellants.

### 3.3. Special Propulsion Applications

**Figure 14:**
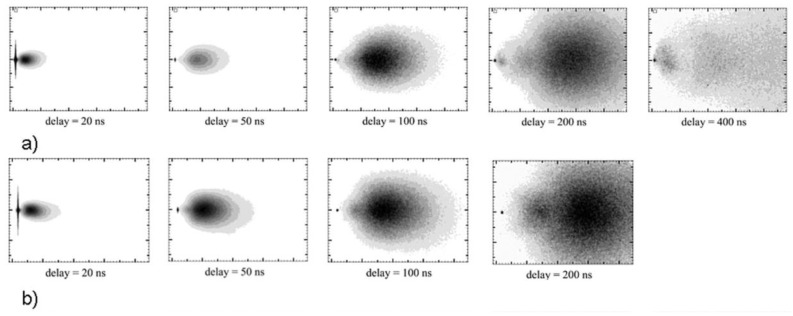
Plasma imaging photographs of the evolution of a plasma plume induced by irradiating the material surface with a laser for: (**a**) GAP+carbon nanoparticles; (**b**) GAP+IR-absorber [100].

Papavlu et al. [100] report an altogether different application for GAP—as an ablative material for use in micro laser plasma thrusters (LPT). Requiring extremely low thrust variation and generally low thrust values (on the order of 100 μN, according to the authors), something that current chemical rocket designs cannot achieve, LPTs generate thrust by ablating propellants to generate directed plasma jets (Figure 14). 

The key qualities of such propellants are low thermal conductivity and highly exothermic decomposition—both being features of energetic polymers, such as GAP or poly (vinyl nitrate) (PVN). Because most such systems are transparent to IR laser wavelengths, they are supplemented with small amounts of IR-absorbers, such as carbon nanoparticles or IR-dyes. Plasma imaging and plasma emission spectroscopic experiments revealed that for GAP, PVN and PVC, the generated plasma plumes contain three domains, with different prevailing propagation velocities, all relatively high, translating into all three propellants being able to produce large amounts of thrust and, as such, being promising materials for LPT applications.

The concept of laser-induced polymer ablations was also approached by Jiao et al. [101], who focused on the methodology of detecting laser-induced plasma plumes and identifying their components. By utilising optical emission spectroscopic imaging, the authors were able to elucidate the mechanisms underlying laser ablation from the three investigated polymers—polyethylene, poly(oxymethylene) and glycidyl azide polymer. Interestingly, when the experiments were conducted in an ultra-low oxygen pressure environment, the polymers were prone to deflagration. When oxygen was substituted for inert argon, this was avoided and significantly lower energy coupling efficiency values were found for polyethylene than for the other two polymers, which in turn showed similar values, despite GAP being an energetic polymer and poly(oxymethylene) not being considered one.

## 4. Applications as an Energetic Binder for Polymer-Bonded Explosives

As has been mentioned in this review, the issue of designing and selecting binders for explosive formulations is a complex issue. Despite GAP being an example of a well-investigated system, it is still crucial to develop and fine-tune its properties and key parameters for novel applications, as well as to examine its behaviour in particular formulations.

ε-2,4,6,8,10,12-Hexanitro-2,4,6,8,10,12-hexaazaisowurtzitane (CL-20) is a small molecule, well known for its high stability (both thermal and chemical), favourable energetic properties and its excellent ability to carry a stable detonation wave, evidenced by reports of very low achievable critical diameters for this explosive [102]. CL-20 is typically used in composite explosives, acting as the energetic filler, along with non-energetic binders. Many formulations based on CL-20 have been reviewed in the past decade by Nair et al. [103], encompassing the use of various such binders.

Gołofit and Zyśk [102] continue investigations into this area, utilising thermal analysis to test the compatibility of CL-20 and several binders (HTPB, butadiene-acrylonitrile-acrylic acid terpolymer, GAP, poly(NIMMO)). Interestingly, the Authors utilise standard procedures (according to STANAG 4147) alongside their modifications, in order to both showcase the effects of any deviation from the set procedures on the result of the evaluation (Table 2) and to provide an insight into the processes taking place in the investigated samples. Although the authors found that CL-20 was incompatible with butadiene-acrylonitrile-acrylic acid terpolymer, GAP, poly(NIMMO) and possibly incompatible with HTPB, their studies revealed that the decomposition of CL-20 is a two stage process.

A similar study was performed by Huang et al. [104] for dihydroxylammonium- 5,5’-bistetrazole-1,1’-diolate (TKX-50, Figure 12), a novel energetic material, which exhibits both low sensitivity and high detonation performance. In this study, the Authors investigated the compatibility of this material with a range of commonly-used energetic and inert substances: nitrocellulose, a NC/NG mixture (1.25:1 weight ratio), 2,4-dinitroanisole, 2,4,6-trinitrotoluene (TNT), RDX, ammonium perchlorate, hexanitroethane, HMX, CL-20, GAP, hydroxyl-terminated polybutadiene, aluminium powder, boron powder, and centralite. Interestingly, the best results were found for 2,4-dinitroanisole and hexanitroethane, both showing good compatibility; GAP was rated as poorly compatible, but slightly better than NC, while still being a far better binder than either HTPB or NC/NG, whose combinations with TKX-50 were rated as hazardous.

Another study carried out to improve design of highly energetic materials basing on coupling between TKX-50 and polymers was carried out in [90]. In this work, the microscopic behaviour of deformation of TKX-50/polymer binders was analysed by means of non-equilibrium molecular dynamics (NEMD) simulations. Four different materials were used as polymer binders: GAP, poly (glycidyl nitrate) (PGN), poly (ethylene glycol) (PEG) and polytetrahydrofuran (poly-THF). The obtained results indicate significant improvement of the mechanical properties of TKX-50 in the presence of every single polymer, with an order of: poly-THF ≈ PEG > PGN > GAP that is connected with lengthening of the side chains of the investigated polymers. When the mass fraction of the polymer binder raised, being set at the levels of: 1%, 5% and 11%, the mechanical properties improved (i.e., TKX-50 stiffness and hardness significantly lowered). At the mass fraction of polymer binders equal to 11%, the polymer binders in TKX-50/GAP and TKX-50/PGN systems self-crimp on the surface of TKX-50, indicating relatively strong interaction of their molecules with TKX-50. This effect was not observed in the case of poly-THF and PEG.

The report by Guo et al. [105] continues this line of research, detailing the properties of compositions consisting of CL-20 and one of various energetic and non-energetic binders. The Authors identify the cracking of CL-20 during a high temperature solid phase transition (ε-phase to the γ-phase) as a potential sensitising factor and, consequently, focus on the effect of the binders on this transition. Interactions between CL-20 and each of the binders are investigated using IR spectroscopy. Although minor or negligible interactions were found for GAP and polyethylene respectively, the spectra of compositions with other binders (Desmodur N100, polyethylenimine and polyvinylpyrrolidone) were significantly different from their parent systems, indicating strong interactions if not an outright chemical reaction, as suspected in the case of CL-20/desmodur N100. The authors mention utilising both liquid and solid-state 1H NMR spectroscopy to further investigate these interactions, but regretfully, these results are not included in the manuscript. An interesting feature of the manuscript is the temperature-dependent XRD study of the compositions, clearly evidencing any transitions in the materials. This experiment reveals that even strong interactions between the components of the composition can have both a positive and negative effect on the system, as exemplified by the spectral sets recorded for CL-20/polyvinylpirrolidone and CL-20/desmodur N100 respectively, showing an increase and a sharp decrease in the ε/γ-phase transition of CL-20. The results of the above compatibility studies are summarised in Table 3 below.

Another study on the CL-20/binder systems is reported by Yanju et al. [106], who focused on the substitution of non-energetic/low-energetic binders with the more energetic GAP. The authors have investigated composites with different CL-20 contents, finding an 82% CL-20 content to be optimal and thoroughly investigated that type of composite in comparison with pristine CL-20. Expectedly, due to the presence of the polymeric binder, the impact and shock sensitivity of the composite were lowered by a factor of two and a factor of six respectively. Despite this, the composite was found to reliably carry detonation in a number of experiments, with its critical diameter being lower than 0.6 mm, exhibiting a detonation velocity of 7364 m·s^−1^, in contrast to 9500 m·s^-1^ for pristine CL-20, entirely sufficient for applications in small size initiation networks.

An interesting report was published by Bayat et al. [107], who were able to produce CL-20/GAP and CL-20/ethylene-vinyl acetate (EVA) polymer bonded explosive (PBX) nanoparticles, utilising a microemulsion phase. The authors investigated different ratios of CL-20 and the binder, achieving roughly constant nanoparticle sizes of 32 nm and 18 nm for CL-20/EVA and CL-20/GAP respectively, as found by dynamic light scattering measurements.

**Figure 15:**
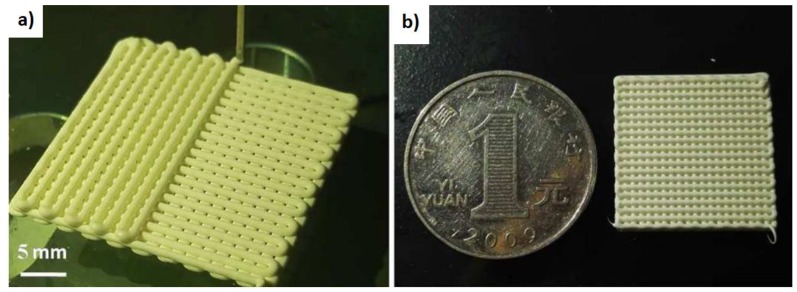
Photos of a CL-20/GAP ink (**a**) being extruded into a multi-layered, three-dimensional grid pattern; and (**b**) constituting the completed 3D printout. Reproduced from [108] with permission from the Royal Society of Chemistry.

Direct ink writing (DIW) is a novel manufacturing method, relying on the extrusion of a filament of “ink” in layered patterns, resulting in a three-dimensional “printout” (Figure 15). Although non-energetic materials are commonly used as inks for such applications, energetic materials can also be used, as shown by Wang et al. [108], who investigated a CL-20/GAP composite as a potential explosive ink for DIW. The composite was tested quite extensively, proving to be non-hygroscopic and exhibiting favourable rheological properties. The key features of the material, however, are its low sensitivity to impact (H_50_ of 37.2 cm, as opposed to 21.1 cm for pristine CL-20) and its ability to maintain detonation even in extremely slim channels, with a critical diameter for the printed composite being reported as less than 0.4 mm.

3,4-Bis(3-nitrofurazan-4-yl)furoxan (DNTF) is a particularly interesting energetic system, as not only does it exhibit thermal stability similar to HMX, while having a burning rate similar to that of CL-20 [109], but also is intensively studied as a melt-cast explosive material. This explosive was used by Chongwei et al. [110] to develop a polymer-bonded explosive (PBX), using GAP as the energetic binder. The authors first investigated the binding ability of GAP towards DNTF, observing the transition from complete bonding to incomplete bonding for a mixture containing 85% DNTF and 10.5% GAP. Working at a slightly lower DNTF/GAP ratio, the authors were able to maximise the performance of the PBX, while ensuring uniformity of the material, as verified by SEM. 

**Figure 16:**
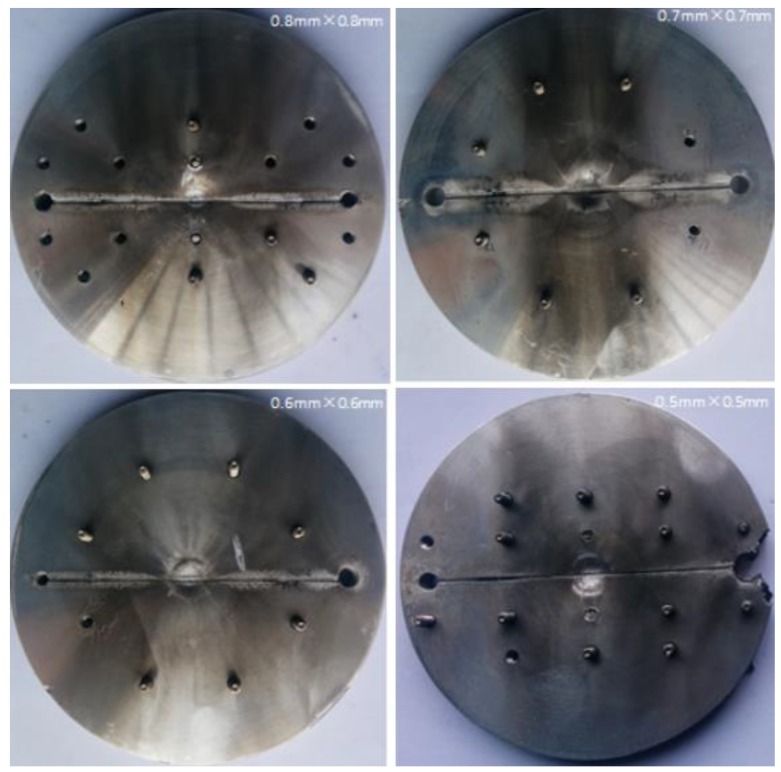
Photos depicting the results of propagation reliability testing for a GAP/DNTF PBX system [110]. The dimensions of the groove intersections in each plate are given in the upper right of each image shown.

The PBX apparently is readily charged into grooves, as required for further investigations, and after a period of curing, which is not given in the manuscript, produces uniform layers, without noticeable cracks or other defects. Investigation of detonation propagation reliability has shown that detonation readily propagates in the PBX, even in grooves as small as 0.5 × 0.5 mm (Figure 16), achieving a detonation velocity as high as 7362 m·s^−1^. Expectedly, the mechanical sensitivity of the PBX is much lower than for pristine DNTF, with the impact sensitivity (h_50_) being 38.3 cm and 25.6 cm respectively. As such, the composition appears to be a very promising replacement for earlier DNTF-based PBXs, particularly for application in highly sophisticated explosive circuits.

Another theoretical study, by Quian et al. [111] deals with the use of a GAP-based polyurethane-urea system for reinforcing TNT, in order to make it less brittle and avert the issue of micro voids in the bulk material caused by thermal expansion of the TNT crystals upon heating. The Authors investigate the inter-molecular interactions to predict the mechanical properties of the two species and their mutual compatibility, concluding that a transition from polyurethane to the GAP-based polyurethane-urea improves its compatibility with TNT and that the mixture is predicted to be more isotropic due to the binder, showing improved mechanical properties.

Manner et al. [112] report an interesting attempt at studying the mechanically-induced changes in PBX samples in situ, during compression, via X-ray computer microtomography. Although the Authors were primarily interested in PBX9501, the explosive (HMX) and binder used in this composition have similar densities and, consequently, show insufficient X-ray contrast. As such, the Authors investigated HMX/HTPB and HMX/GAP systems to obtain an insight into the mechanical changes taking place in a PBX sample during compression. Utilising the in situ method, they were able to observe the occurrence and evolution of a range of deformation and fracture defects, with their type and nature being dependent on binder stiffness and HMX crystal-binder adhesion strength.

Hussein et al. [113] have recently investigated the properties of PBXs using GAP and have compared them with properties reported for pure explosives [114,115] and PBXs using HTPB [116]. The authors investigated PBXs containing 16% (*w*/*w*) GAP supplemented with dioctyl adipate and 84% of one of the following explosives: β-HMX, RDX, ε-CL-20 and cis-1,3,4,6-tetranitro- octahydroimidazo-[4,5-d] imidazole (BCHMX). The key parameters found by the authors and reported in literature have been compiled in Table 4. Interestingly, while the substitution of HTPB with GAP results in slightly less favourable sensitivity parameters, all other key properties are improved significantly. The one exception is seen for PBXs based on ε-CL-20, in whose case lower friction sensitivity is seen for the PBX using GAP rather than the one using HTPB. The Authors followed this work up with a TG and DSC investigation of the interactions of GAP and selected nitroamines, finding that GAP increases their thermal reactivity [117]. This is particularly the case for RDX, as the Authors have observed the decomposition of the GAP/RDX PBX in its molten state.

## 5. Other Applications and Modifications of GAP

**Figure 17:**
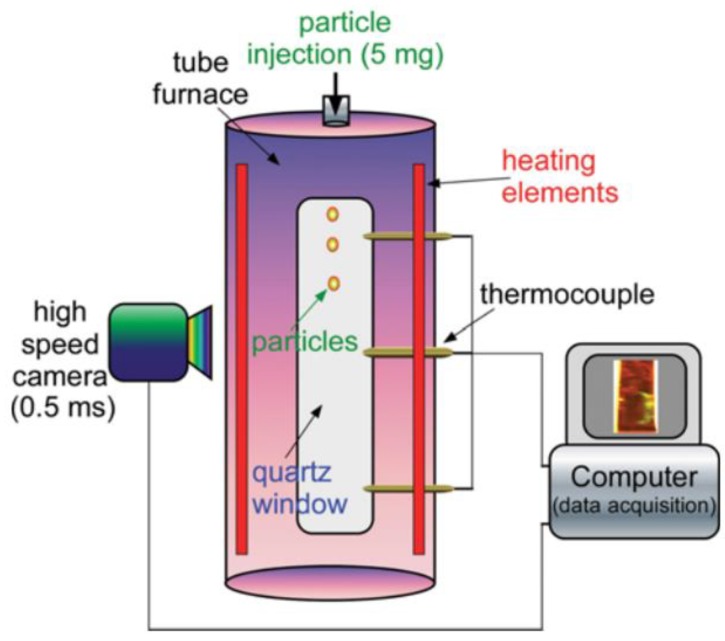
Scheme of the experimental setup for high-speed time-resolved monitoring of the combustion of a particle cloud [118].

Although the vast majority of reports concerning GAP and its derivatives are dedicated to the chemistry and technology of pyrotechnics and explosives, the polymer is versatile enough to be utilised in other areas of application as well. These applications need not even be related to processes or devices that involve combustion and include a wide array of seemingly unrelated fields, such as drug delivery or organic electronics. An interesting report by Shin et al. [118] details the study of the combustion of boron particles that were coated with GAP to improve their combustion profiles. The authors were able to prepare such GAP-coated particles and even decorate them with silver nanoparticles, with the nature and properties of the two types of species being documented in good detail. A novel setup was used to investigate the burn time of a cloud of such nanoparticles (Figure 17), with the course of combustion being followed with a 0.5 ms resolution, using a high-speed camera, revealing an extremely rapid 37.5 ms burn time.

**Figure 18:**
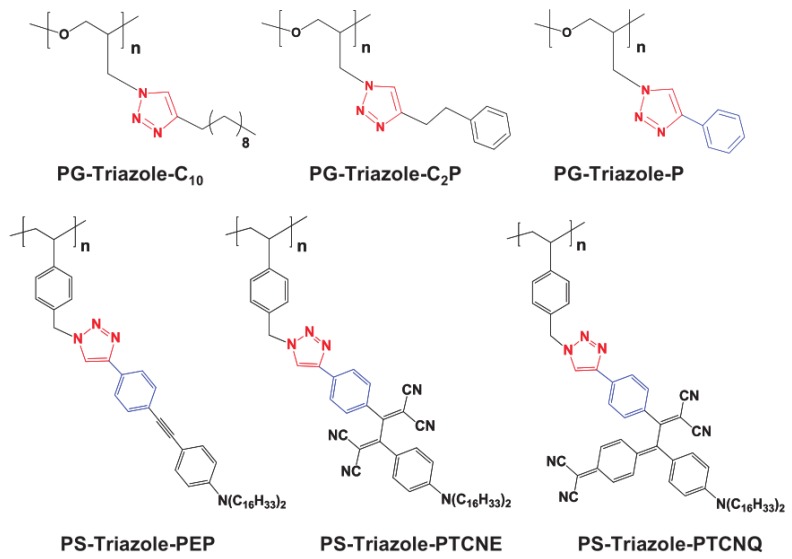
Chemical structures of GAP-derived and poly(4-azidomethylstyrene)-derived polymer brushes for applications in organic electronics [119].

The triazole functionality, introduced into GAP through the reaction of its azide groups with alkynes has also attracted interest as a reactive site for the introduction of side chains and preparation of polymer brushes [119]. By introducing π-conjugated systems as the side chains (Figure 18), the authors were able to obtain dielectric polymers with a potential application in electrical memory devices. Not only did the polymers show high ON/OFF currents, important for such applications, but could work as either p- or n-type memory, making this transformation of GAP particularly promising.

**Figure 19:**
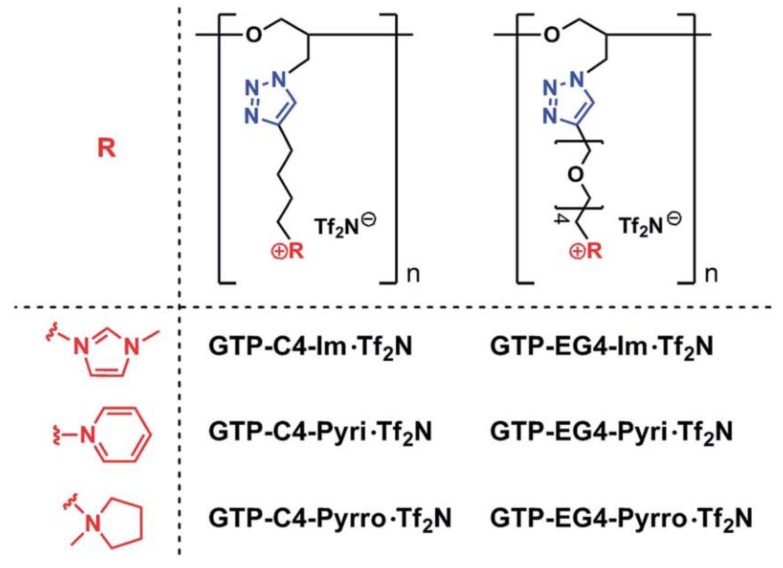
Chemical structures of GAP-derived ionic liquid polymers. The symbol Tf_2_N denotes the bis (trifluoromethanesulfonyl) imide moiety. Reproduced from [63] with permission from the Royal Society of Chemistry.

A different approach to this method involves using alkynated derivatives of ionic liquid materials instead of the π-conjugated side systems as substrates for the azide-alkyne reaction [63]. In this case, by selecting the type of ionic moiety and length of the spacer between it and the produced triazole moiety (Figure 19), the authors were able to control the properties of their poly (ionic liquids) to a significant extent, producing materials, whose DC conductivities could be altered by more than one order of magnitude.

Ikeda continued the above work, in an attempt to introduce different types of substituents via the azide-alkyne reaction [120]. An interesting feature of this report is that by using prop-2-ynoic acid and but-2-ynedioic acid esters rather than species, in which the alkyne group was attached to an alkyl chain, it was possible to forego the copper catalyst used in the authors’ previous work. The use of ester groups also grants the resultant modified polymer the ability to undergo hydrolysis and possible further modification. Another aspect worth noting is that the use of but-2-ynedioic acid esters allows producing derivatives bearing a disubstituted triazole ring (formed in the azide-alkyne reaction). Although the same is theoretically possible to achieve by using non-terminal alkynes, no such reports have been found for GAP.

Polymer-peptide conjugates are therapeutic agents that exhibit the biological activity of peptides, while utilising the properties of polymers to improve their delivery as well as their pharmacokinetic and pharmacodynamics properties. Initially, linear polyethers were used for this purpose, with the peptides being introduced at the terminal functional groups, allowing only very low loading capacities. As such, research efforts shifted towards polymers bearing side chains that could be functionalised to enable linkage with peptides and affording significantly higher loading capacities. One of the methods of producing such side-chain-peptide linkage relies on the azide-alkyne reaction, making GAP a potential substrate for such applications, as reported by O’Connor et al. [121]. The authors report successful synthesis of the GAP-peptide conjugate and show that linking the peptide with different polymers does not affect its biological activity adversely.

An interesting study was performed by Lemos and Bohn [122], dealing with a new polyester polyol binder, Desmophen 2200, and the effect of several plasticisers, including GAP, on its glass transition temperature. Unfortunately the Authors included neither a structure of the Desmophen binder nor referred to one of several commercially available Desmophen 2200 types, making the exact structure of the macromolecule unclear. The use of GAP as an energetic plasticiser rather than an energetic binder by itself is also curious, particularly so due to it being combined with a non-energetic binder rather than with another energetic binder. Nevertheless, after curing a Desmophen 2200 sample, containing 35% (*w*/*w*) GAP with Desmodur N3400 (a [NCO]/[OH] ratio of 0.85 is reported to have been used for all samples, although it is not known if that calculation included the hydroxyl groups present in GAP as well), a material exhibiting a glass transition temperature between −50.4 °C (for a deformation frequency of 0.1 Hz) and −41.4 °C (frequency of 30 Hz) was obtained. Although a strict comparison between the dynamic mechanical analysis and DSC results is impossible without standardisation between the two works, it is worth noting that Zhang [49] reported a DSC-determined glass transition temperature of −45.3 °C for GAP cured with Desmodur N100 (**CI2**), appearing to be similar to the abovementioned, more elaborate system. Unfortunately, although the Authors indicate that the binder-plasticiser system is targeted for application in composite rocket propellants, no tests involving an actual propellant formulation are reported.

Sprayable pyrotechnic formulations are an extremely sophisticated subject with many potential applications. A number of such formulations designed as pyrotechnic biocides, including one involving GAP, are reported by Oxley et al. [123]. The formulations were designed to act by releasing heat and free iodine, both being strongly biocidic. Due to this dual nature, an uncommon oxidising/reducing agent was employed, in the form of a mixture of calcium iodate, Ca(IO_3_)_2_, and aluminium. This system was used in a number of matrices—both urethane foam and nitrocellulose-in-acetone systems were used, yielding different consistencies after drying. Interestingly, while a GAP-based matrix proved to be more energetic than a nitrocellulose-based one, it was also found to have a reduced iodine output. Even though a system containing 50% GAP and 50% Ca(IO_3_)_2_ could readily be painted on a surface, it was found to be sensitive to impact and as such, the Authors recommended the use of nitrocellulose-based formulations.

An interesting application of GAP, as a protective and energetic layer for Al nanoparticles, is reported by Zeng et al. [124]. The authors designed the in situ grafting of GAP on Al as a measure for combating the passivation of Al, a phenomenon that greatly limits the usability of nano-scale dispersions of Al. An additional advantage of this approach is that after grafting GAP onto them, the Al nanoparticles become hydrophobic, as evidenced by the sharp increase of the wetting angle (from approx. 20° for untreated Al to above 140° for Al-graft-GAP). Although the authors did not perform such an investigation, it is likely that Al-graft-GAP-based propellants will be less sensitive to exposure to humidity than Al-based propellants.

**Scheme 6:**
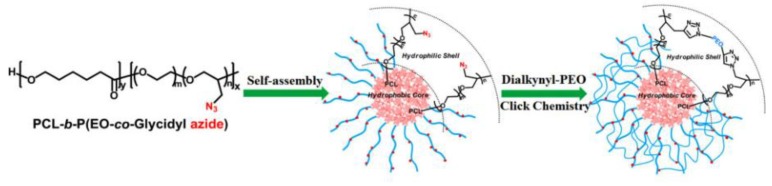
Schematic representation of the self-assembly of a GAP copolymer into micelles and curing of the micelles via the azide-alkyne reaction of GAP functional groups [123].

The report by Liu and Gan [125] highlights the versatility of both the synthetic pathway used to prepare GAP and the polymer itself; instead of using a homopolymer for the azidation stage, the Authors opted to use poly(ethylene oxide-co-epichlorohydrin) as the substrate. The resultant poly (ethylene oxide-co-glycidyl azide) was then used to prepare its block copolymer with ε-caprolactone, in the aim to produce a material capable of self-assembly into micelles. These micelles were then cured using alkynyl-terminated poly (ethylene oxide), forming triazole linkages in a reaction with the azide groups of GAP (Scheme 6), and evaluated as potential materials for drug delivery systems.

## 6. Summary and Conclusions

Of the 76 experimentally investigated systems based on GAP or its derivatives, 25 deal purely with GAP, whether it is cured, modified or used “as is” in testing. 42 works report investigations of mixtures of GAP and other species; of those, many deal with the preparation of GAP-based copolymers through curing of mixtures of polymers and/or monomers, whereas others focus on the use of GAP as a binder. Only five works directly deal with GAP-containing copolymers, prepared through copolymerisation of a mixture of co-monomers and subsequent azidation of the copolymer. Four more works deal with derivatives of GAP or species similar to GAP, e.g., GAP grafted onto carbon nanotubes and a glycidyl nitramine polymer respectively.

If considered in terms of curing agents and methodology, 35 works focus on the use of classical isocyanate curing agents (reaction with terminal hydroxyl groups), ten attempt curing GAP with multifunctional alkynes (reaction with the azide groups) and only three works adopt a mixed methodology, employing both isocyanate and alkyne curing agents, either simultaneously or consecutively. A further five works use alkynes to introduce additional functional groups into GAP instead of curing the polymer. Carboxyl acid chlorides and mesyl-terminated species are also used for curing GAP, represented each by a single report. The remaining 18 reports use no curing agents, either due to the focus of the study, such as preparation of GAP-based xerogels or study of GAP thinners for improving its viscosity, or due to the fact that some species require no curing, such as the abovementioned glycidyl nitramine polymer, which is a solid.

In terms of mechanical properties of cured GAP and its derivatives, both cured and investigated post synthesis there is large variation, with the reported Young’s modulus values ranging from 0.1 MPa up to even 174 MPa. A similar discrepancy is also observed for reported tensile strength and elongation at break values. Although the differences in the chemical structure of these polymers and copolymers can account for much of this variation, we suspect a very significant effect of the staggering variety of methodologies used for investigating the mechanical properties of these systems.

This is particularly likely, as authors report wildly differing properties for GAP cured using the same curing agent, even when a similar reactive group ratio is used. To exemplify, for GAP cured by **CA3**, at a ratio of 1:1, Sonawane [19] and Hagen [28] report Young’s modulus values of 0.16 and 0.55 MPa, tensile strength values of 0.11 and 0.34 MPa and elongation at break values of 73.2 and 68% respectively. A similar case is seen for GAP cured by an isocyanate curing agent. In this case, Xu [47]cured GAP using **CI1**, at a isocyanate to hydroxyl molar ratio of 1:1, reporting a tensile strength of 2.4 MPa and a 101% elongation at break. Utilising the same curing agent, at an even higher ratio (**CI1**/GAP of approx. 1.4:1), Ma [70] reports a tensile strength of 0.21 MPa and a 286% elongation at break.

In light of the abovementioned discrepancies, a comparison of the mechanical properties of different GAP-based systems would best be realised if the properties of each unique polymer or copolymer were related to the properties of a “baseline” GAP, investigated using the same methodology. Although some authors implement this methodology, not only does this comprise a minority of published works, but also various cured GAP derivatives are chosen as the baselines.

Taking the above into consideration, the best comparison that can be made is internally, among the results achieved in a particular report, showing only qualitatively how a particular modification affects the mechanical properties of the investigated system. As such, we have compiled Table 5, grouping the reported mechanical properties by the works they were reported in rather than, as would be expected, by the chemical structure of the main polymeric species and the curing agent.

Both GAP and its derivatives, as energetic materials, should also be considered in terms of their sensitivity and combustion/detonation parameters, whether as the binder itself or as the binder in an energetic formulation. As such, we have compiled Table 6 grouping the reported sensitivity and energetic properties.

The numerous approaches to the structural modification of GAP allow us to tailor its various properties in an extremely wide range. Although some approaches can yield very favourable properties, the effective cost of producing such binders should also be taken into account, as more cost-efficient systems can become more commonly used, even despite exhibiting slightly worse performance.

The addition of GAP to various explosives and propellant formulations typically results in limiting their sensitivity, particularly to mechanical stimuli, as even cured GAP is a relatively soft material, capable of absorbing and dispersing the strain caused by the application of such stimuli. It is worth noting that, apart from its properties as a structural scaffold, GAP or cured GAP still contains many functional groups, which can interact with a variety of species, e.g., promoting adhesion as a result. Simultaneously, while bestowing such favourable features or improving the properties of the base material/composition, GAP (and other energetic binders) has the advantage over more traditional binders in the fact that it only slightly affects the combustion/detonation parameters.

It is also worth mentioning that apart from its “typical” uses in conjunction with energetic materials/compositions, the ability to functionalise GAP either through the hydroxyl or azide groups leads to a number of various, seemingly unexpected applications, such as in ionic liquids or even organic electronics.

## Figures and Tables

**Table 1:** Typical properties of commercially available GAP-polyol [7].

Property [Units]	Value
Viscosity at 25 °C [Pa·s]	12 ^a,b^
Density [g/cm^3^]	1.3
Hydroxyl equivalent weight [g/mol]	2000 ^c^
Functionality [-OH groups per molecule]	2.5 ÷ 3 ^c^
Solubility	Most organic solvents, but not water, lower alcohols or aliphatic hydrocarbons
Impact sensitivity test (Bureau of Explosives)	0/10 at 9.04 J ^d^ neat
Friction sensitivity test (Bureau of Explosives)	0/10 at 444.8 N ^e^

^a^ converted from centipoise in the original source, using the relation 1 cP = 1 × 10^−3^ Pa·s, ^b^ in comparison, the viscosity of water at this temperature is approx. 8.94 × 10^−4^ Pa·s; ^c^ based on the supplied information, an average molecular weight of 5000 ÷ 6000 g/mol is estimated; ^d^ converted from inch-pounds in the original source, using the relation 1 J = 8.85 inch-pounds; ^e^ converted from pounds in the original source, using the relation 1 N = 0.2248 pounds.

**Table 2:** Results of compatibility tests for a mixture of CL-20 and poly(NIMMO), performed in different conditions [102].

Decomposition Temperature of CL-20 (T_A_)	Heating Rate	Experimental Pan	Decomposition Temperature of Mixture (T_AB_)	Compatibility (Criterion: Value of T_AB_-T_A_)
500 K ^a^	2 K/min	w/pinhole	474 K	<−20K, incompatible
499 K	2 K/min	Hermetic	474 K	<−20K, incompatible
505 K	10 K/min	w/pinhole	486 K	−19 K, possibly incompatible
529 K	10 K/min	Hermetic	491 K	<−20 K, incompatible

^a^ Compatibility test conditions in accordance with STANAG 4147 standard guidelines.

**Table 3:** Summary of DSC-based compatibility study results.

Components ^a^		Heating Rate [K/min]	Type of Experimental Pan	Δt ^b^ [k]	Authors’ Compatibility Evaluation	Ref.
CL-20	poly(NIMMO)	2	w/pinhole	−26	incompatible	[102]
CL-20	PBAN			−30	incompatible	
CL-20	HTPB			−20	possibly incompatible	
CL-20	GAP			−31	incompatible	
TKX-50	2,4-Dinitroanisole	10	open, N_2_ atmosphere	−2.03	A–B (compatible/slightly sensitised)	[104]
TKX-50	TNT			−11.47	C (sensitised)	
RDX	TKX-50			−17.64	D (hazardous)	
TKX-50	HMX			−3.89	B (slightly sensitised)	
CL-20	TKX-50			−8.17	C (sensitised)	
TKX-50	Centralite			−10.82	C (sensitised)	
NC	TKX-50			−9.85	C (sensitised)	
NC+NG ^c^	TKX-50			−16.9	D (hazardous)	
TKX-50	Ammonium chlorate(VII)			−8.77	C (sensitised)	
Hexanitro-ethane	TKX-50			+8.04	A (compatible)	
TKX-50	Al			−8.01	C (sensitised)	
TKX-50	B			−17.61	D (hazardous)	
TKX-50	GAP			−6.16	C (sensitised)	
TKX-50	HTPB			−68.4	D (hazardous)	
CL-20 ^d^	Polyethylene			+3.9	-	[105]
CL-20 ^d^	GAP			−8.4	-	
CL-20 ^d^	Desmodur N100			−31.8	-	
CL-20 ^d^	Polyvinylpyrrolidone			−0.6	-	
CL-20 ^d^	Polyethylenimine			+2.9	-	

^a^ The component in the left-hand component column was treated as the “main”, as its decomposition peak maximum (T_A_) occurred at a higher temperature than the analogous maximum of the component in the right-hand component column; ^b^ The value ΔT was calculated as ΔT = T_AB_ − T_A_, where T_AB_ is the temperature of the first decomposition peak maximum of the mixture of both components; ^c^ 1.25:1 (*w*/*w*) mixture of nitrocellulose and nitroglycerine; d The decomposition temperature of the other component, in its pristine state, was not measured; hence, CL-20 was used as the point of reference for calculating ΔT.

**Table 4:** Properties of pure explosives, PBXs using HTPB and PBXs using GAP.

Explosive	BCHMX			β-HMX			RDX			ε-CL20		
Binder	-	HTPB	GAP	-	HTPB	GAP	-	HTPB	GAP	-	HTPB	GAP
Density [g/cm^3^]	1.79	1.56	1.62	1.90	1.57	1.64	1.76	1.52	1.59	1.98	1.63	1.73
Detonation velocity [m/s]	8650	7746	8292	9100	7812	8384	8750	7526	8074	9473	8167	8676
Detonation pressure [GPa]	33.9	21.2	28.6	38.0	21.3	28.4	32.1	20.1	26.2	41.7	23.7	32.1
Heat of explosion [kJ/kg]	6447	5744	6658	6075	5453	6297	6085	5453	6152	6455	5786	6559
Heat of combustion [J/g]	9124	13,798	11,789	9485	14,118	11,893	9522	14,162	12,011	8311	13,255	10,922
Impact sensitivity [J]	3.2	9.6	7.7	6.4	15.2	11.2	5.6	14.6	11.5	4.1	10.8	8.4
Friction sensitivity [N]	88	322	294	95	>360	338	120	>360	>360	69	214	247

**Table 5:** Properties of energetic systems presented in the reviewed works. The annotated and more detailed version of this table is included in the supplementary materials as Table S1.

Main Component	Curing Agent	Young’s Modulus MPa	Tensile Strength MPa	Elongation at Break %	Notes/Ratios of Reagents	Ref.
GAP	CA1	5.36	2.53	47.6	N3/alkyne 2:1	[17]
		0.65	0.33	81.6	N3/alkyne 3:1	
GAP	CA2	174.1	13.1	~28	CA2/GAP 5:1	[18]
		9.1	4.5	81.7	CA2/GAP 3:1	
GAP	CA3	0.07	0.08	147	GAP/CA3 0.9:1	[19]
		0.16	0.11	73.2	GAP/CA3 1:1	
		0.25	0.12	48.3	GAP/CA3 1.1:1	
			0.18	47.78	GAP/CA3 1.2:1	
		0.53	0.19	38.5	GAP/CA3 1.3:1	
		0.72	0.20	27	GAP/CA3 1.4:1	
		4.17	1.04	28.11	GAP/CA3 2.5:1	
GAP	CI2	0.21	0.25	148.5	not given	[20]
	CA3	8.22	3.58	47.78		
	2-nitroderivative of CA3	5.72	1.83	35.84		
GAP	CA4	1.52	0.21	47.6	alkyne/azide 1.2:1	[22]
		3.60	0.41	25.4	alkyne/azide 2:1	
PAMMO		0.89	0.41	66.4	alkyne/azide 1.2:1	
		2.56	0.67	37	alkyne/azide 2:1	
Acyl-GAP	CA2	-	2.69	86	10% CA2	[23]
		-	1.28	464	Acyl-GAP + CA2/HTPB + CI1 0:1	
		-	2.69	86	Acyl-GAP + CA2/HTPB + CI1 1:0	
		-	5.26	318	Acyl-GAP + CA2/HTPB + CI1 1:1	
MWCNT-HTPB + MWCNT-Acyl-GAP	CI1, CI2	-	8.17	312	NCO/OH = 0.8; 50% Acyl-GAP	[24]
HTPB+GAP		-	5.89	359	NCO/OH = 1; 50% GAP	[25]
GAP	CA9	3.23	0.80	28.3	Azide/alkyne 1:2.5	[26]
	CA10	7.44	1.77	36.3	Azide/alkyne 1:2.5 + 1% MWCNT	
	CA11	6.33	1.41	19.7	Azide/alkyne 1:2.5 + 1% MWCNT-COOH	
GAP	dual isocyanate/CA6	1.68	-	-	NCO/OH 1.1:1; GAP/dipolarophile 100:1 (*w*/*w*)	[27]
		3.29	-	-	NCO/OH 1.1:1; GAP/dipolarophile 100:1,5 (*w*/*w*)	
		4.69	-	-	NCO/OH 1.1:1; GAP/dipolarophile 100:2 (*w*/*w*)	
		7.64	-	-	NCO/OH 1.1:1; GAP/dipolarophile 100:5 (*w*/*w*)	
	dual isocyanate/CA5	3.07	-	-	NCO/OH 1.1:1; GAP/dipolarophile 100:1.5 (*w*/*w*)	
		5.42	-	-	NCO/OH 1.1:1; GAP/dipolarophile 100:2 (*w*/*w*)	
		7.6	-	-	NCO/OH 1.1:1; GAP/dipolarophile 100:5 (*w*/*w*)	
GAP	CI2	0.18	0.26	161	NCO/OH 1:0.8	[28]
		0.47	0.5	122	NCO/OH 1:1	
		1.09	0.7	69	NCO/OH 1:1.2	
	CI1, CI2 (1:1)	0.082	0.23	285	NCO/OH 1:1	
		0.315	0.52	192	NCO/OH 1:1.2	
		0.6	0.76	147	NCO/OH 1:1.4	
	CA3	0.55	0.34	68	CA3/GAP: 1:1	
		1.24	0.57	48	CA3/GAP: 1.3:1	
	CA3, CI1	0.71	0.6	95	CA3/GAP: 0.5:1; NCO/OH 1:1	
		1.28	0.85	72	CA3/GAP: 0.7:1; NCO/OH 1:1	
	CA7, CI1	0.57	0.79	157	CA7/GAP: 0.5:1; NCO/OH 1:1	
		1.11	1.2	117	CA7/GAP: 0.7:1; NCO/OH 1:1	
GAP/Al/NH_4_ClO_4_/HMX/Bu-NENA	-	1.48	0.26	45.6	Blank sample	[36]
		1.86	0.29	37.9	BindAgent1 0.1%	
		2.17	0.35	36.7	BindAgent1 0.2%	
		2.26	0.36	33.1	BindAgent1 0.3%	
		1.57	0.3	35.2	BindAgent2 0.1%	
		1.59	0.36	33.8	BindAgent2 0.2%	
		1.8	0.41	32.7	BindAgent2 0.3%	
		1.85	0.32	45.2	BindAgent3 0.1%	
		2.07	0.39	44.2	BindAgent3 0.2%	
		2.1	0.39	43.1	BindAgent3 0.3%	
GAP	toluene diisocyanate	-	1.6	1041	R = NCO/OH. R = 1.6	[37]
FGAP	CI2	-	1.5	81.6	NCO/OH = 1	[47]
GAP		-	0.66	51.1		
GAP	CI1	-	2.4	101	NCO/OH = 1	[50]
FGAP		-	5.52	162.8		
ETPE+NC	-	-	7.5	490	NC 0%	[52]
		-	8.9	485.6	NC 10%	
		-	7.8	110.5	NC 20%	
		-	8.7	96.8	NC 30%	
		-	13.3	45.1	NC 40%	
		-	18.2	28.9	NC 50%	
PBAMO/GAP	toluene diisocyanate + 1,4-butanediol	-	2.55	217	TDI+BDO = 30%	[53]
GAP/P(EO-co-THF)	CI1, CI2 NCO/OH = 1,2:1	-	0.662	212.4	Flexible polyether: 0%( *w*/*w*)	[54]
		-	0.694	237.4	Flexible polyether: 5% (*w*/*w*)	
		-	0.824	279.6	Flexible polyether: 10% (*w*/*w*)	
		-	0.857	280.7	Flexible polyether: 15% (*w*/*w*)	
		-	0.933	284.1	Flexible polyether: 20% (*w*/*w*)	
		-	0.953	289.9	Flexible polyether: 25% (*w*/*w*)	
		-	0.986	296.1	Flexible polyether: 30% (*w*/*w*)	
GAP/PAO	CI1, CI2 NCO/OH = 1,2:1	-	0.662	212.4	Flexible polyether: 0% (*w*/*w*)	
		-	0.753	256.7	Flexible polyether: 5% (*w*/*w*)	
		-	0.885	260.4	Flexible polyether: 10% (*w*/*w*)	
		-	1.633	271.4	Flexible polyether: 15% (*w*/*w*)	
		-	1.917	276.1	Flexible polyether: 20% (*w*/*w*)	
		-	2.528	330.1	Flexible polyether: 25% (*w*/*w*)	
		-	3.512	435.4	Flexible polyether: 30% (*w*/*w*)	
GAP-based PU	CI1	0.12	0.21	286	Alkyne/azide = 0/1; 2.27 mmol CI1 for 1.62 mmol GAP	[70]
DNPMP (n=1)/GAP-based PU		0.13	0.27	293	Alkyne/azide = 0.1/1	
		0.06	0.43	505	Alkyne/azide = 0.3/1	
		1.05	1.71	390	Alkyne/azide = 0.5/1	
DNPMB (n=2)/GAP-based PU		0.09	0.3	402	Alkyne/azide = 0.1/1	
		0.08	0.41	539	Alkyne/azide = 0.3/1	
		0.34	1.21	461	Alkyne/azide = 0.5/1	
PDNP (n=1)/GAP-based PU		0.1	0.32	425	Alkyne/azide = 0.1/1	
		0.15	0.55	638	Alkyne/azide = 0.3/1	
		6.06	2.9	687	Alkyne/azide = 0.5/1	
BDNP (n=2)/GAP-based PU		0.09	0.28	421	Alkyne/azide = 0.1/1	
		0.11	0.57	574	Alkyne/azide = 0.3/1	
		3.2	1.67	684	Alkyne/azide = 0.5/1	
GAP doped NC and NG propellants	-	-	44.97	7.314	30% GAP/NC	[84]
		-	44.93	7.311	30% GAP/NC	
		-	44.82	7.727	30% GAP/NC	
		-	37.71	37.4	40% GAP/NC	
		-	37.37	36.94	40% GAP/NC	
		-	38.42	36.57	40% GAP/NC	
		-	29.92	75.78	50% GAP/NC	
		-	30.16	74.37	50% GAP/NC	
		-	29.45	74.9	50% GAP/NC	
		-	31.38	35.11	40% GAP/NG	
		-	31.05	35.17	40% GAP/NG	
		-	31.61	35.29	40% GAP/NG	
GAP doped propellants	CI2	5.26	0.72	26.3	0% GAP/−40 °C	[85]
	CI3	7.18	1.18	30.2	10% GAP/−40 °C	
	CI4	7.17	1.16	29.8	20% GAP/−40 °C	
	CI5	3.02	0.41	34.9	0% GAP/+20 °C	
	CI6	3.81	0.54	52.5	10% GAP/+20 °C	
	CI7	3.71	0.55	52.1	20% GAP/+20 °C	
	CI8	2.14	0.4	39.5	0% GAP/+50 °C	
	CI9	2.23	0.5	52.4	10% GAP/+50 °C	
	CI10	2.2	0.45	52.6	20% GAP/+50 °C	
GAP (21%) doped propellants	CI2	4.71	0.29	18.9	Propellant 1	[87]
		4.53	0.26	14.7	Propellant 2	
		2.25	0.22	24.4	Propellant 3	

**Table 6:** Sensitivity and explosive properties of energetic systems presented in the reviewed works. The annotated and more detailed version of this table is included in the supplementary materials as Table S2.

Investigated System [details]	Sensitivity/Other Properties	Ref.
GAP [commercial]	Impact: >9.04 J Friction: >444.8 N	[7]
MWCNT-HTPB + MWCNT-Acyl-GAP [cured with CI1 and CI2, NCO/OH = 0,8; 50% Acyl-GAP]	Impact: >40 J Friction: >360 N Electrostatic discharge: >5 J	[24]
Propellants based on MWCNT-HTPB [cured with CI1 and CI2]	Impact: 3–4 J Friction: 40–50 N	
Propellants based on MWCNT-HTPB + MWCNT-Acyl-GAP [cured with CI1 and CI2, NCO/OH = 0.8; 50% Acyl-GAP]	Impact: 4,5 J Friction: 210 N	
GAP-co-azidoTHF	Impact: 0% Friction: 0% Electrostatic discharge: 0.181 J (E_50_)	[43]
GAP [cured with CI4]	Impact: >40 J Friction: >360 N	[51]
Poly (2,2,2-trifluoro-ethoxymethyl epoxy-r-glycidyl azide) [cured with C1, NCO/OH = 1]	Impact: >129 cm (H_50_)	[52]
GAP [cured with C1, NCO/OH = 1]	Impact: 42.2 cm (H_50_)	
GAP [cured with CI3]	Impact: 7 J Friction: >360 N	[71]
BAMP [cured with diisocyanatoethane]	Impact: 40 J Friction: >360 N Electrostatic discharge: 1.5 J	
BAMP [cured with CI3]	Impact: 40 J Friction: >360 N	
DNPD [cured with diisocyanatoethane]	Impact: 40 J Friction: >360 N	
DNPD [cured with CI3]	Impact: 40 J Friction: >360 N Electrostatic discharge: 1.5 J	
Esters of BAMP	Impact: >40 J Friction: >360 N	[61,63]
Esters of DNPD	Impact: >40 J Friction: >360 N	
Bis (2,3-diazidopropoxy) alkanes GlyN dimer	Friction: >360 N	[71]
GNAP	Impact: 40 J Friction: >360 N V_DET_: 7165 m/s (theoretical)	[71]
NC/NG modified with GAP [0% GAP, powder mixture]	Impact: 6.92 J (E_50_)	[85]
NC/NG modified with GAP [10% GAP, powder mixture]	Impact: 7.1 J (E_50_)	
NC/NG modified with GAP [20% GAP, powder mixture]	Impact: 9.05 J (E_50_)	
NC/NG modified with GAP [30% GAP, powder mixture]	Impact: 9.34 J (E_50_)	
NC/NG/GAP-based propellant [0% GAP in powder mixture, 4.0–5.2% “free” GAP in propellant slurry]	Impact: 6.86 J (E_50_)	[94]
NC/NG/GAP-based propellant [10% GAP in powder mixture, 4.0–5.2% “free” GAP in propellant slurry]	Impact: 8.29 J (E_50_)	
NC/NG/GAP-based propellant [20% GAP in powder mixture, 4.0–5.2% “free” GAP in propellant slurry]	Impact: 10.23 J (E_50_)	
NC/NG/GAP-based propellant [30% GAP in powder mixture, 4.0–5.2% “free” GAP in propellant slurry]	Impact: 13.11 J (E_50_)	
RDX (95) coated with NC (3) and GAP (2)	Impact: 40.74 cm (H_50_)	
RDX	Impact: 23.40 cm (H_50_)	
RDX [pure]	Impact: 12.8 cm (H_50_)	[96]
RDX/GAP [blend cured with CI5, NCO/OH = 1.5, RDX = 40% *w*/*w*]	Impact: 15.59 cm (H_50_)	
RDX/GAP [nanocomposite cured with CI5, NCO/OH = 1.5, RDX = 40% *w*/*w*]	Impact: 30.2 cm (H_50_)	
εCL-20/GAP [82% Cl-20, 14% GAP (Mn = 3380, with a hydroxyl value of 0.646 mol/g), 1.5% toluene diisocyanate, 2,5% others, cured with toluene diisocyanate]	Impact: 38.2 cm (H_50_) Shock sensitivity: 7.74 mm (card gap) V_DET_: 7364 m/s Critical diameter: <0.6 mm	[107]
CL-20 [pure]	Impact: 15.9 cm (H_50_) Shock sensitivity: 42.11 mm (card gap) V_DET_: 9500 m/s	
CL-20/GAP [85% CL-20, cured with CI2. GAP Mn = 4000, with a hydroxyl value of 1.412 mmol/g]	Impact: 37.2 cm (H_50_) Critical diameter: <0.4 mm	[109]
CL-20	Impact: 21.1 cm (H_50_)	
GAP/DNTF [DNTF 85%, GAP (Mn ≈ 3300) 11%, toluene diisocyanate 1.6%, others 2.4%]	Impact: 38.3 cm (H_50_) Friction: 0% V_DET_: 7362 m/s Critical diameter: <0.5 mm	[111]
DNTF (2–3 μm)	Impact: 25.6 cm (H_50_) Friction: 13%	
DNTF (15–20 μm)	Impact: 25.0 cm (H_50_) Friction: 12%	
HMX	Impact: 25.7 cm (H_50_) Friction: 100%	

## Supplementary Materials

The following are available online, Table S1: Mechanical properties of reviewed energetic systems (annotated), Table S2: Sensitivity and energetic properties of reviewed energetic systems (annotated).
