# Supplementary material for: Glycidyl Azide Polymer and its Derivatives-Versatile Binders for Explosives and Pyrotechnics: Tutorial Review of Recent Progress
Source: Molecules. 2019 Dec 6;24(24):4475. doi: 10.3390/molecules24244475 (PMC6943510; doi:10.3390/molecules24244475)
Supplement: Supplementary file 1 [file molecules-24-04475-s001.zip › Table S1.pdf]

Main component(s)	Curing agent(s)	Young's modulus [MPa]	Tensile strength [MPa]	Elongation at break [%]	Reagent ratios / notes	Comments / methodological notes	Ref.
GAP	CA1	5.36	2.53	47.6	N3/alkyne 2:1	10mm wide strips, tensile rate 50mm/min, CMT6503 universal testing machine (SANS, Shenzhen, China)	17
		0.65	0.33	81.6	N3/alkyne 3:1		
GAP	CA2	174.1	13.1	~28	CA2/GAP 5:1	10mm wide strips, tensile rate 50mm/min, CMT6503 universal testing machine (SANS, Shenzhen, China)	18
		9.1	4.5	81.7	CA2/GAP 3:1		
GAP	CA3	0.07	0.08	147	GAP/CA3 0,9:1	Universal testing machine (UTM) model: Hounsfield, H25KS, UK [dumbbell shaped test specimens, at ambient temperature (27 8C) and rate of testing 50 mm/min	19
		0.16	0.11	73.2	GAP/CA3 1:1		
		0.25	0.12	48.3	GAP/CA3 1,1:1		
			0.18	47.78	GAP/CA3 1,2:1		
		0.53	0.19	38.5	GAP/CA3 1,3:1		
		0.72	0.20	27	GAP/CA3 1,4:1		
		4.17	1.04	28.11	GAP/CA3 2,5:1		
GAP	CI2	0.21	0.25	148.47	not given	Universal Testing Machine((Hounsfield, H25KS)	20
	CA3	8.22	3.58	47.78	not given		
	2-nitroderivative of CA3	5.72	1.83	35.84	not given		
GAP	CA4	1.52	0.21	47.6	alkyne / azide 1,2:1	INSTRON 6022 universal testing machine with a cross-head speed of 20 mm/min and a mean value of four replicates was taken	22
		3.60	0.41	25.4	alkyne / azide 2:1		
pAMMO		0.89	0.41	66.4	alkyne / azide 1,2:1		
		2.56	0.67	37	alkyne / azide 2:1		
Acyl-GAP	CA2	-	2.69	86	10% CA2	Dumbbell shape samples, universal testing machine Instron-6022, Shimadzu Co., Ltd., constant rate of 100 mm/min and mean values of five samples were taken	125
		-	1.28	464	Acyl-GAP+CA2/HTPB+CI1 0:1		
		-	2.69	86	Acyl-GAP+CA2/HTPB+CI1 1:0		
		-	5.26	318	Acyl-GAP+CA2/HTPB+CI1 1:1		
MWCNT-HTPB + MWCNT-Acyl-GAP	CI1, CI2	-	8.17	312	NCO/OH=0,8; 50% Acyl-GAP	Instron-6022, Shimadzu Co., Ltd, dumbbell-shape specimens, constant rate of 100 mm/min, based on ASTM D638	24
HTPB+GAP		-	5.89	359	NCO/OH=1; 50% GAP		25
GAP	CA9	3.23	0.80	28.3	Azide / alkyne 1:2,5	CMT6503 universal testing machine (SANS, Shenzhen, China) at a speed of 50 mm/min. Five samples were tested for each composite and the average value was presented graphically	26
	CA10	7.44	1.77	36.3	Azide / alkyne 1:2,5 + 1% MWCNT		
	CA11	6.33	1.41	19.7	Azide / alkyne 1:2,5 + 1% MWCNT-COOH		
GAP	dual isocyanate/CA6	1.68	-	-	NCO/OH 1,1:1; GAP / dipolarophile 100:1 (w/w)	Dumbbell-shaped specimens (measuring 68.58x8.7x12.6 for the composite propellants) were determined using a universal testing machine (Model 5567, 1597 * 909 * 700 mm, Instron) at a crosshead rate of 50 mm/min at room temperature	27
		3.29	-	-	NCO/OH 1,1:1; GAP / dipolarophile 100:1,5 (w/w)		
		4.69	-	-	NCO/OH 1,1:1; GAP / dipolarophile 100:2 (w/w)		
		7.64	-	-	NCO/OH 1,1:1; GAP / dipolarophile 100:5 (w/w)		
	dual isocyanate/CA5	3.07	-	-	NCO/OH 1,1:1; GAP / dipolarophile 100:1,5 (w/w)		
		5.42	-	-	NCO/OH 1,1:1; GAP / dipolarophile 100:2 (w/w)		
		7.6	-	-	NCO/OH 1,1:1; GAP / dipolarophile 100:5 (w/w)		
GAP	CI2	0.18	0.26	161	NCO/OH 1:0,8	810 MTS (Material Testing System), using microtensile specimens prepared according to ASTM D1708. All tensile testing was conducted at room temperature, using a crosshead speed of 50 mm/min	28
		0.47	0.5	122	NCO/OH 1:1		
		1.09	0.7	69	NCO/OH 1:1,2		
	CI1, CI2 (1:1)	0.082	0.23	285	NCO/OH 1:1		
		0.315	0.52	192	NCO/OH 1:1,2		
		0.6	0.76	147	NCO/OH 1:1,4		
	CA3	0.55	0.34	68	CA3/GAP: 1:1		
		1.24	0.57	48	CA3/GAP: 1,3:1		
	CA3, CI1	0.71	0.6	95	CA3/GAP: 0,5:1; NCO/OH 1:1		
		1.28	0.85	72	CA3/GAP: 0,7:1; NCO/OH 1:1		
	CA7, CI1	0.57	0.79	157	CA7/GAP: 0,5:1; NCO/OH 1:1		
		1.11	1.2	117	CA7/GAP: 0,7:1; NCO/OH 1:1		
GAP/Al/NH4ClO4/HMX/BuNENA	-	1.48	0.26	45.6	Blank sample	Dumbbell specimens, Instron-6022, Shimadzu Co., Ltd.), crosshead speed of 100 mm/min, room temperature. Five specimens were tested for each sample	36
		1.86	0.29	37.9	BA1 0,1%		
		2.17	0.35	36.7	BA1 0,2%		
		2.26	0.36	33.1	BA1 0,3%		
		1.57	0.3	35.2	BA2 0,1%		
		1.59	0.36	33.8	BA2 0,2%		
		1.8	0.41	32.7	BA2 0,3%		
		1.85	0.32	45.2	BA3 0,1%		
		2.07	0.39	44.2	BA3 0,2%		
		2.1	0.39	43.1	BA3 0,3%		
GAP	toluene diisocyanate	-	1.6	1041	R = NCO/OH, R=1,6	Instron 26022 Universal testing machine in accordance with GB/T528-1998	37
FGAP	CI2	-	1.5	81.6	NCO/OH = 1	AG-X Plus testing machine (Shimadzu, Japan), with a tensile rate of 50 mm/min. The test films were cut into dumbbell-shaped samples with a width of 20mm and the distance between testing marks was 30 mm. The test strips were kept at 0% humidity for 7 days before measurement. A mean value of five replicates from each film was taken.	47
GAP		-	0.66	51.1			
GAP	CI1	-	2.4	101	NCO/OH = 1	Shimadzu AG-X Plus universal testing machine (Shimadzu, Japan) with a tensile rate of 50 mm/min. The copolyurethane networks were cut into dumbbell-shaped specimens (dimensions: 35 mm x 6 mm x 2 mm) and kept at 0% humidity for 7 days before measurement. A mean value of five replicates from each film was taken	50
FGAP		-	5.52	162.8			
ETPE+NC	-	-	7.5	490	NC 0%	Instron 6022 (Shimadzu Co. Ltd, Japan), constant strain rate of 100mm/min, room temperature. The dimensions of the dumb-bell samples were 20mm (neck area length) × 4mm (width) × 2mm (thickness). The reported values were calculated as average values from five specimens of each sample	52
		-	8.9	485.6	NC 10%		
		-	7.8	110.5	NC 20%		
		-	8.7	96.8	NC 30%		
		-	13.3	45.1	NC 40%		
		-	18.2	28.9	NC 50%		
PBAMO/GAP	toluene diisocyanate + 1,4-butanediol	-	2.55	217	TDI+BDO = 30%	Instron-6022 (Shimadzu Co., Ltd.), strain rate of 100 mm/min, 25 *C.The dimensions of the samples were 20 mm (neck area length) 9 4 mm (width) 9 2 mm (thickness). We usually take 5 samples and use the method of averaging the results	53
GAP/P(EO-co-THF)	CI1, CI2 NCO/OH = 1,2:1	-	0.662	212.4	Flexible polyether: 0 %(w/w)	Dumbbell specimens, tensile testing machine [AGS-X, Shimadzu Co., Ltd. (Kyoto, Japan)], crosshead speed of 100mm/min, at 25 and -40*C. Five specimens were tested for each sample to obtain average values. Samples were measured after conditioning the specimen at a given temperature for a minimum duration of 4h	54
		-	0.694	237.4	Flexible polyether: 5 %(w/w)		
		-	0.824	279.6	Flexible polyether: 10 %(w/w)		
		-	0.857	280.7	Flexible polyether: 15 %(w/w)		
		-	0.933	284.1	Flexible polyether: 20 %(w/w)		
		-	0.953	289.9	Flexible polyether: 25 %(w/w)		
		-	0.986	296.1	Flexible polyether: 30 %(w/w)		
GAP/PAO	CI1, CI2 NCO/OH = 1,2:1	-	0.662	212.4	Flexible polyether: 0 %(w/w)		
		-	0.753	256.7	Flexible polyether: 5 %(w/w)		
		-	0.885	260.4	Flexible polyether: 10 %(w/w)		
		-	1.633	271.4	Flexible polyether: 15 %(w/w)		
		-	1.917	276.1	Flexible polyether: 20 %(w/w)		
		-	2.528	330.1	Flexible polyether: 25 %(w/w)		
		-	3.512	435.4	Flexible polyether: 30 %(w/w)		
GAP-based PU	CI1	0.12	0.21	286	Alkyne/azide = 0/1; 2,27mmol CI1 for 1,62 mmol GAP	texture analyzer (TA-HD+1500, Stable Micro Systems Ltd.)	
DNPMP (n=1)/GAP-based PU		0.13	0.27	293	Alkyne/azide = 0.1/1		
		0.06	0.43	505	Alkyne/azide = 0.3/1		

	CI1	1.05	1.71	390	Alkyne/azide = 0.5/1	with a 50 kgf load cell, TA-51 needle jig, and software (Texture Expert, Stable Micro Systems Ltd.). The probe moving velocity was 0.8 mms ¢1 . The tensile test samples were cut from the cast film with the specification (DIN-53504, type (S2)). All tensile strengths, ultimate elongations and initial moduli reported were the averages of three measured values	70
DNPMB (n=2)/GAP-based PU		0.09	0.3	402	Alkyne/azide = 0.1/1		
		0.08	0.41	539	Alkyne/azide = 0.3/1		
		0.34	1.21	461	Alkyne/azide = 0.5/1		
PDNP (n=1)/GAP-based PU		0.1	0.32	425	Alkyne/azide = 0.1/1		
		0.15	0.55	638	Alkyne/azide = 0.3/1		
		6.06	2.9	687	Alkyne/azide = 0.5/1		
BDNP (n=2)/GAP-based PU		0.09	0.28	421	Alkyne/azide = 0.1/1		
		0.11	0.57	574	Alkyne/azide = 0.3/1		
		3.2	1.67	684	Alkyne/azide = 0.5/1		
GAP doped NC and NG propellants	-	-	44.97	7.314	30% GAP / NC	Instron-6022, Shimadzu Co., Ltd., China, constant rate of 100 mm/min, at room temperature	84
		-	44.93	7.311	30% GAP / NC		
		-	44.82	7.727	30% GAP / NC		
		-	37.71	37.4	40% GAP / NC		
		-	37.37	36.94	40% GAP / NC		
		-	38.42	36.57	40% GAP / NC		
		-	29.92	75.78	50% GAP / NC		
		-	30.16	74.37	50% GAP / NC		
		-	29.45	74.9	50% GAP / NC		
		-	31.38	35.11	40% GAP / NG		
		-	31.05	35.17	40% GAP / NG		
		-	31.61	35.29	40% GAP / NG		
GAP doped propellants	CI2	5.26	0.72	26.3	0% GAP / -40°C	Instron 4505 tensile tester. Cured propellants were cut into slices, from which JANNAF dog bones were stamped. The tests were carried out at temperatures of -40, 20 and 50 degrees Celsius with 100 mm/min cross-head speed	85
	CI3	7.18	1.18	30.2	10% GAP / -40°C		
	CI4	7.17	1.16	29.8	20% GAP / -40°C		
	CI5	3.02	0.41	34.9	0% GAP / +20°C		
	CI6	3.81	0.54	52.5	10% GAP / +20°C		
	CI7	3.71	0.55	52.1	20% GAP / +20°C		
	CI8	2.14	0.4	39.5	0% GAP / +50°C		
	CI9	2.23	0.5	52.4	10% GAP / +50°C		
	CI10	2.2	0.45	52.6	20% GAP / +50°C		
GAP (21%) doped propellants	CI2	4.71	0.29	18.9	Propellant 1	810 MTS (Material Testing System) according to the procedure of STANAG 4506. Dog bone samples (crosshead speed=50 mm/min)	87
		4.53	0.26	14.7	Propellant 2		
		2.25	0.22	24.4	Propellant 3		
