# Supplementary material for: Glycidyl Azide Polymer and its Derivatives-Versatile Binders for Explosives and Pyrotechnics: Tutorial Review of Recent Progress
Source: Molecules. 2019 Dec 6;24(24):4475. doi: 10.3390/molecules24244475 (PMC6943510; doi:10.3390/molecules24244475)
Supplement: Supplementary file 1 [file molecules-24-04475-s001.zip › Table S2.pdf]

| Main component(s) | Curing agent(s) | Impact sensitivity [J] | Friction sensitivity [N] | Electric spark discharge sensitivity [J] | Ratios of the components / main component and curing agent | Comments and methodology notes | Ref. |
|---|---|---|---|---|---|---|---|
| GAP (commercial) | - | >9,04 | >444,8 | - | - | Bureau of Explosives: Impact 0/10 80 inch-pound (9,04J) (http://www.utec-corp.com/wp-content/uploads/2017/10/BOE.pdf Test method 1011 as detailed in MIL-STD 1751A) friction 0/10 100 pounds (444,8 N) | 7 |
| MWCNT-HTPB + MWCNT-Acyl-GAP (NCO/OH=0,8; 50% Acyl-GAP) | CI1, CI2 | >40 | >360 | >5 | - | | 24 |
| Propellants based on MWCNT-HTPB | CI1, CI2 | 3-4 | 40-50 | - | - | 80% solid loadings, NH4ClO4, Al, exact composition not given | |
| Propellants based on MWCNT-HTPB + MWCNT-Acyl-GAP (NCO/OH=0,8; 50% Acyl-GAP) | CI1, CI2 | 4.5 | 210 | - | - | 80% solid loadings, NH4ClO4, Al, exact composition not given | |
| GAP-co-azidoTHF | - | 0% | 0% | 0.181 | - | Impact sensitivity: QJ2913-1997 standard; Friction sensitivity: QJ1469-1988 standard, Electrical spark discharge sensitivity: QJ 3039-1998 standard (E50%) | 41 |
| GAP | CI4 | >40 | >360 | - | "cured GAP" + butanediol + N-(2-cyanoethyl)diethanolamine \| GAP = soft segment, rest = hard segment = 30% \| ETPE X consists of X% ethanolamine in butanediol/ethanolamine mixture | Insensitive for all ETPE X types. | 49 |
| poly(2,2,2-trifluoro-ethoxymethyl epoxy-r-glycidyl azide) | CI1 | >129cm | - | - | NCO/OH = 1 | H50 / drop hammer impact sensitivity test for energetic polymer was developed. The weight of the hammer was 3 kg and the drop height was between 0–1.29 m | 50 |
| GAP | CI1 | 42,2cm | - | - | NCO/OH = 1 | | |
| GAP | CI3 | 7.00 | >360 | - | - | Impact and friction sensitivity tests were carried out according to STANAG 4489 18 and STANAG 4487 modified instructions using a BAM (Bundesanstalt f€ ur Materialforschung) drop hammer and friction tester. Electrostatic sensitivities were measured with a OZM small scale electrostatic discharge tester. | 58 |
| BAMP | diisocyanatoethane | 40.00 | >360 | 1.5 | - | | |
| BAMP | CI3 | 40.00 | >360 | - | - | | |
| DNPD | diisocyanatoethane | 40.00 | >360 | - | - | | |
| DNPD | CI3 | 40.00 | >360 | 1.5 | - | | |
| Esters of BAMP | - | >40 | >360 | - | - | Impact and friction sensitivity tests were carried out according to STANAG 4489 and STANAG 4487 modified instructions using a BAM (Bundesanstalt für Materialforschung) drop hammer and friction tester. Compounds used as plasticisers for GAP | 60 |
| Esters of DNPD | - | >40 | >360 | - | - | | |
| Bis(2,3-diazidopropoxy)alkanes | - | - | >360 | - | - | BAM friction apparatus was used | 62 |
| GlyN dimer | - | - | >360 | - | - | | |
| GNAP | - | 40 | >360 | - | Theoretical Vdet (7165m/s), from EXPLO 5 V6.02 \| ESD sensitivity is mentioned but no values are given | Impact and friction sensitivity tests were carried out according to STANAG 4489 and STANAG 4487 modified instructions using a BAM (Bundesanstalt für Materialforschung) drop hammer and friction tester. Electrostatic sensitivities were measured with a OZM small scale electrostatic discharge tester | 71 |
| NC/NG modified with GAP | - | 6.92 | - | - | 0%GAP in the GAP/NC/NG powder | The standard impact tester was used with exchangeable anvil; the amount of tested substance was 50 mm 3 ; drop hammers of 2 kg mass were used. The sensitivity observed was expressed as the drop energy, Edr, vs. percentage of initiation. Only the 50 % probability of initiation is used in this article (E50) | 85 |
| NC/NG modified with GAP | - | 7.1 | - | - | 10%GAP in the GAP/NC/NG powder | | |
| NC/NG modified with GAP | - | 9.05 | - | - | 20%GAP in the GAP/NC/NG powder | | |
| NC/NG modified with GAP | - | 9.34 | - | - | 30%GAP in the GAP/NC/NG powder | | |
| NC/NG modified with GAP + GAP + other components = propellant slurry | - | 6.86 | - | - | 0%GAP in the GAP/NC/NG powder and 4.0-5.2 "free" GAP added to the mixture | | |
| | - | 8.29 | - | - | 10%GAP in the GAP/NC/NG powder and 4.0-5.2 "free" GAP added to the mixture | | |
| | - | 10.23 | - | - | 20%GAP in the GAP/NC/NG powder and 4.0-5.2 "free" GAP added to the mixture | | |
| | - | 13.11 | - | - | 30%GAP in the GAP/NC/NG powder and 4.0-5.2 "free" GAP added to the mixture | | |
| RDX (95% w/w) coated with NC (3%) and GAP (2%) | - | 40,74cm | - | - | - | The impact sensitivity was surveyed by a home-built type-12 drop hammer apparatus. The test was carried out by the Chinese National Military Standard GJB-772A-97, standard method 601.2 [17]. The samples (35+/-1 mg) were subjected to an impact of a 2.500+/-0.002 kg hammer at various heights using an up-and-down method. The critical drop height of 50% explosion probability H50 | 93 |
| RDX | - | 23,40cm | - | - | - | | |
| RDX (pure) | - | 12,8cm | - | - | RDX = 100% | The impact sensitivity was measured by 5 kg standard drop-hammer [15, 16], and the charge weight was 35 mg. DetectedbyChina OrdnanceIndustriesbooster chargeperfor-mance testing center at room temperature. Firstly, the samples were placed at the bottom of the drop hammer. Secondly, 5 kg weight drop hammer was released when it was promoted to a certain height. Thirdly, the hammer hit the target on the ground, resulting in an explosive. An average value of impact sensitivity was obtained based on the results from three tests. The impact sensitivity value represents the height from which the dropping of a 5-kg weight will result in an explosion in 50 % of the trials. | 96 |
| RDX/GAP (blend) | CI5, NCO/OH = 1,5 | 15,59cm | - | - | RDX = 40% | | |
| RDX/GAP (nanocomposite) | CI5, NCO/OH = 1,5 | 30,2cm | - | - | RDX = 40% | | |
| Cl-20/GAP | toluene diisocyanate | 38,2cm | - | - | CL-20 (ε type) was provided by Liaoning Qingyang Chemical Industry Co.Ltd. (Liaoning, China); GAP, Mn = 3380, with a hydroxyl value of 0.646 mol/g was from Luzhou North Chemical Industry Co. Ltd \| 82% Cl-20, 14% GAP, 1,5% toluene diisocyanate, 2,5% others \| shock sensitivity 7,74mm (card gap) \| Vdet (measured) = 7364m/s \| critical diameter <0,6mm | The drop weight was 5.000 ± 0.005 kg, and sample mass was 35 ± 1 mg. The result is represented with the characteristic height (H 50 ). The larger the value of H 50 , the lower the impact sensitivity (National Military Standard of China, China Weapon Industry Corporation). The shock sensitivity test assembly is shown in Fig. 2. The experimental principle is that a shock wave was generated by the explosion of standard donor explosive column. Then the shock wave was attenuated by the card gap of polymethyl methacrylate. Finally, the attenuated shock wave acted on the test sample. The shock sensitivity and its safety were evaluated by the thickness of the card gap. | 106 |
| Cl-20 (pure) | - | 15,9cm | - | - | shock sensitivity 42,11mm (card gap) \| Vdet 9500m/s | | |
| Cl-20/GAP (85% Cl-20) | CI2 | 37,2cm | - | - | Used for DIW (direct ink writing) \| CL-20 explosive (purity=99.6 %, particle size 20-50 μm) synthesised \| Glycidyl azide polymer binder (GAP, Number-average molecular weight, M n = 4000, with a hydroxyl value of 1.412 mmol/g, Liming Research Institute of Chemical Industry), Desmodur N100 commercial \| critical diameter <0,4mm | The impact sensitivity was determined by using a BAM fall hammer apparatus. A 30 ± 1 mg sample was placed steel anvils and was hit by a 2 ± 0.05 kg hammer. The environmental temperature and humidity are at 23 °C and 20 %. The results are represented with the characteristic height ( H 50 ) | 108 |
| Cl-20 | - | 21,1cm | - | - | - | | |
| GAP/DNTF | toluene diisocyanate | 38,3cm | 0% | - | DNTF was supplied by Gansu YinGuang Chemical Industry Co. Ltd. GAP resin with a molecular weight close to 3300 was produced by Luzhou North Chemical Industry Co. Ltd. \| DNTF 85%, GAP 11%, | In the impact sensitivity test, an ERL type 12 drop hammer apparatus was used in which the mass of the drop weight was 2.500 ± 0.005 kg and the sample mass was 35 ± 1 mg. All tests were performed at room temperature. Friction sensitivity was measured according to the explosion probability method | 109 |

| | | | | | | | |
|---|---|---|---|---|---|---|---|
| DNTF (2-3 μm) | - | 25,6cm | 13% | - | toluene diisocyanate 1,6%, others 2,4% \| critical diameter < 0,5mm, Vdet = 7362m/s | of GJB-772 using a Swing Friction Sensitivity Tester | |
| DNTF (15-20 μm) | - | 25,0cm | 12% | - | | | |
| HMX | - | 25,7cm | 100% | - | | | |
